# Post pandemic fatigue: what are effective strategies?

**DOI:** 10.1038/s41598-022-13597-0

**Published:** 2022-06-11

**Authors:** Ziyue Yuan, Salihu Sabiu Musa, Shu-Chien Hsu, Clara Man Cheung, Daihai He

**Affiliations:** 1 Department of Civil and Environmental Engineering, Hong Kong Polytechnic University, Hung Hom, Hong Kong; 2 Department of Applied Mathematics, Hong Kong Polytechnic University, Hung Hom, Hong Kong; 3 Department of Mathematics, Kano University of Science and Technology, Wudil, Nigeria; 4 Department of Mechanical, Aerospace and Civil Engineering, School of Engineering, University of Manchester, Manchester, UK

**Keywords:** Risk factors, Infectious diseases

## Abstract

Recurrent updates in non-pharmaceutical interventions (NPIs) aim to control successive waves of the coronavirus disease 2019 (COVID-19) but are often met with low adherence by the public. This study evaluated the effectiveness of gathering restrictions and quarantine policies based on a modified Susceptible-Exposed-Infectious-Hospitalized-Recovered (SEIHR) model by incorporating cross-boundary travellers with or without quarantine to study the transmission dynamics of COVID-19 with data spanning a nine-month period during 2020 in Hong Kong. The asymptotic stability of equilibria reveals that the model exhibits the phenomenon of backward bifurcation, which in this study is a co-existence between a stable disease-free equilibrium (DFE) and an endemic equilibrium (EE). Even if the basic reproduction number (${\mathscr {R}}_{0}$) is less than unity, this disease cannot be eliminated. The effect of each parameter on the overall dynamics was assessed using Partial Rank Correlation Coefficients (PRCCs). Transmission rates (i.e., $\beta _1$ and $\beta _2$), effective contact ratio $a_6$ between symptomatic individuals and quarantined people, and transfer rate $\theta _3$ related to infection during quarantine were identified to be the most sensitive parameters. The effective contact ratios between the infectors and susceptible individuals in late July were found to be over twice as high as that in March of 2020, reflecting pandemic fatigue and the potential existence of infection during quarantine.

## Introduction

The coronavirus disease 2019 (COVID-19) outbreak led to a global pandemic in early 2020^1^. The disease has reached almost every country in the world. Since then, many countries such as the USA, England, and Italy have experienced several waves of the epidemic^2^. By March 2021, the total number of COVID-19 cases exceeded 119.2 million, including more than 2.64 million deaths globally^1^. Its spread has also left economies and businesses counting the costs as governments struggle with instituting and enforcing various non-pharmaceutical intervention (NPI) measures (e.g. social distancing, face coverings, and mandatory quarantine of inbound travellers) to slow down the spread of the virus. Although the recent rollout of severe acute respiratory syndrome coronavirus 2 (SARS-CoV-2) vaccines has raised hopes that the pandemic is nearing an end, identifying the duration of immunity, i.e., how long a person is protected after being vaccinated, could take several years of monitoring and research^3^. If immunity declines before herd immunity-when a large portion of the population of an area achieves immunity-previously vaccinated individuals will become susceptible to infection again. Under these circumstances, implementing effective NPIs remains critical to controlling the spread of COVID-19^4^.

Yet according to the World Health Organisation (WHO), as the pandemic has continued to persist, the NPIs implemented in many countries have caused an increase in “pandemic fatigue”, that is, demotivation about following recommended or required measures to protect themselves and others from the virus^4^. It becomes a growing challenge for governments to find effective ways to handle this fatigue and reinvigorate public vigilance. To guide governments in the planning and implementing NPIs, WHO developed a framework of policy recommendations in late 2020 with four key strategies^4^. One of the strategies highlighted the importance of collecting and using evidence for targeted, tailored, and effective policies, interventions, and communication. In line with this strategy, infectious disease modelling techniques, aptly named compartment models, have been used to provide insights into creating more targeted NPIs to control COVID-19 transmission.

Indeed, compartment models have been used for a long time to study disease transmission dynamics and gain insight into how diseases spread, which can help in devising prevention and control measure. Compartment models are formulated based on dividing populations into mutually-exclusive compartments representing disease status using the Kermack-McKendrick framework^5^. For example, Wu et al.^6^ calculated the reproduction number, ${\mathscr {R}}_{0}$, of COVID-19 as 2.68 via the use of the Susceptible-Exposed-Infectious-Removed (SEIR)-based model. Tang et al.^7^ employed a dynamic model to assess the efficiency of travel restrictions, and Lin et al.^8^ examined transmission trends and the effects of NPIs on the dynamics of COVID-19 spread by using an SEIR-based model.

Many previous studies have found that human mobility by transportation such as air^9^, rail^10^, or public transit^11^ have contributed to epidemic diffusion. The use of quarantines for members of the general public has been studied to combat the spread of respiratory diseases^6,12–14^. By employing a modified Susceptible-Exposed-Infectious-Hospitalized-Recovered (SEIHR) model to assess the transmission dynamics of SARS-CoV-2, this study extends previous work by incorporating inbound travellers with and without quarantine into the studied population in order to better understand how those NPIs affect transmission. Some basic qualitative properties of the model are analyzed, such as the basic reproduction number ${\mathscr {R}}_{0}$ and stability of the equilibria. The model is fitted using data on Hong Kong to show the trends characterizing the spread of the disease. Hong Kong was selected because it is a densely populated city with a higher risk and speed of COVID-19 transmission^15^.

During this pandemic, a government policy stringency assessment system, which was developed by Oxford University and partners, and uses 20 indicators to generate scores ranging from 0 to 100, gave Hong Kong an average score of 56 in terms of its response to COVID-19^16^. As of 14th April 2021, Hong Kong has implemented strict quarantine policies for travellers and close contacts of infected persons, during which time it recorded 11,612 cases and 209 deaths and still met a fourth wave of COVID-19 infections^17^. As a highly densely-populated city, it is especially critical for Hong Kong to be able to implement feasible measures for controlling the spread of COVID-19.

## Results

### Modelling

**Figure 1:**
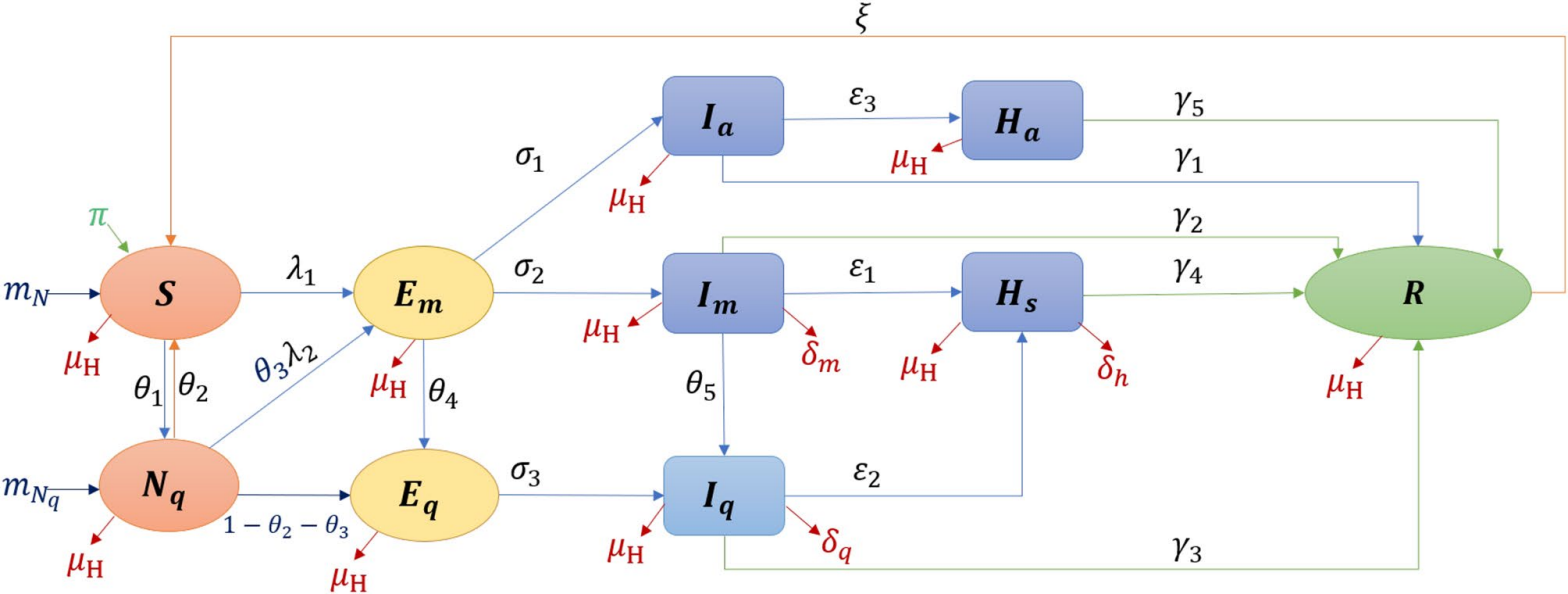
Susceptible-Exposed-Infectious-Hospitalised-Recovered (SEIHR) model.

A modified compartmental model is developed to overcome the limitations of only considering well-mixed homogeneous populations in the previous well-established compartment model. The newly proposed model can divide the population into ten groups based on transmission characteristics (shown in Fig. 1). An individual may progress from being susceptible (*S*) to being exposed ($E_{m}$) to being asymptomatic/symptomatic ($I_{a}$ or $I_{m}$) to being hospitalized ($H_{a}$ or $H_{s}$) to having recovered (*R*), and can be quarantined while being susceptible ($N_{q}$), exposed ($E_{q}$) or infectious ($I_{q}$). Pre-symptomatic, symptomatic and asymptomatic individuals are all contagious to susceptible individuals under effective contact, i.e., when an already infected individual is in contact with another individual who thus may become infected as well. In our model, the duration of the transmission spans from being pre-symptomatic to being hospitalized. The amount of virus in the bodies of infected individuals during incubation increases over several days (which is assumed in our study to be three days) before symptom onset^18^. It is often not easy to study the transmission onset time, as it is difficult to know who infected whom exactly when. This study assumed that each exposed individual was pre-symptomatic and contagious. Due to a lack of symptoms, both exposed individuals $E_m$ and asymptomatic infectious individuals $I_a$ may come in contact with both susceptible individuals with ($N_q$) and without (*S*) quarantine through outside movement, in household settings, or during provision of daily necessities by volunteers. The force of infection, which is the rate at which individuals become infected per unit time^19^, is shown in Eq. (2). Each COVID-19 confirmed case can continue to shed the virus to others up to and including during hospitalization. Owing to reinfection^20^ and virus mutations^21^, an infected individual’s convalescence period may end without lifelong immunity-a recovered individual transfer from *R* to *S* by a reinfection rate ξ. Considering that many countries suffered from several waves due to imported cases and frequent virus mutations, and that COVID-19 might become a seasonal disease^22^, the proposed model is designed with a dynamic population by natural birth and death, mortality from COVID-19, and cross-boundary (in and out of a given territory or country) human mobility.

### Mathematical analysis

#### Steady states

Disease extinction and persistence^23^ are determined by the stability of the disease-free equilibrium (DFE) and the endemic equilibrium (EE) of the model (1). Under the locally asymptotical stability in this system (i.e., DFE), applying the next-generation method^24^ to the equations in the model (1) (see SI.2), the basic reproduction number ${\mathscr {R}}_{0}$ (i.e., the average number of secondary cases caused by each infectious individual) in Eq. (5) is contributed by three groups (${\mathscr {R}}_{0}= R_{E_{m}} + R_{I_{m}} + R_{I_{a}}$). The interpretation of ${\mathscr {R}}_{0}$ is shown in Table 1. To be more specific, $R_{E_{m}}={(a_{4}\beta _{2}\theta _{3}+a_{1}\beta _{1})}/{q_{1}}$, which is caused by exposed individuals with outside movement, $R_{I_{m}}={(a_{6}\beta _{2}\theta _{3}+a_{3}\beta _{1})\sigma _{2}}/{q_{1}q_{4}}$, which is caused by symptomatic infectious individuals with outside movement, and $R_{I_{a}}={(a_{5}\beta _{2}\theta _{3}+a_{2}\beta _{1})\,\sigma _{1}}/{q_{1}q_{3}}$, which is caused by asymptomatic individuals with outside movement, can be explained by the force of infection Eq. (2).

**Table 1:** Interpretation of the basic reproduction number $R_{0}$.

Section	Equation	Interpretation
$R_{E_{m}}$	$\frac{a_{4}\beta _{2}\theta _{3}+a_{1}\beta _{{1}}}{q_{1}}$	Numerator: the infections produced by $E_{m}$; Denominator: the population transferring out of $E_{m}$
$R_{I_{m}}$	$\frac{(a_{6}\beta _{2}\theta _{3}+a_{3}\beta _{1})\sigma _{2}}{q_{1}q_{4}}$	$a_{6}\beta _{2}\theta _{3}+a_{3}\beta _{1}$: the infections produced by $I_{m}$; $\sigma _{2}/q_{1}q_{4}$: the net population left in $I_{m}$ among population from $E_{m}$
$R_{I_{a}}$	$\frac{(a_{5}\beta _{2}\theta _{3}+a_{2}\beta _{1})\sigma _{1}}{q_{1}q_{3}}$	$a_{5}\beta _{2}\theta _{3}+a_{2}\beta _{1}$: the infections produced by $I_{a}$; $\sigma _{1}/q_{1}q_{3}$: the net population left in $I_{a}$ among population from $E_{m}$

The pandemic is still evolving with several resurgences globally. The possible coexistence of DFE with a stable EE is explored. A global asymptotic stability exists, which corresponds to positive solutions to Eq. (19) (proven in SI.3). The backward bifurcation (BB) phenomenon has been shown to exist when the classical epidemiological requirement of having ${\mathscr {R}}_{0}<1$ is no longer sufficient for effective control of COVID-19 infections. Substituting the force of infection Eq. (17) and global asymptotically points (in SI.2) into the total population at EE Eq. (18), there are 32 scenarios that reflects the plausibility of BB phenomenon (see Table 4).

#### Sensitivity analysis

**Figure 2:**
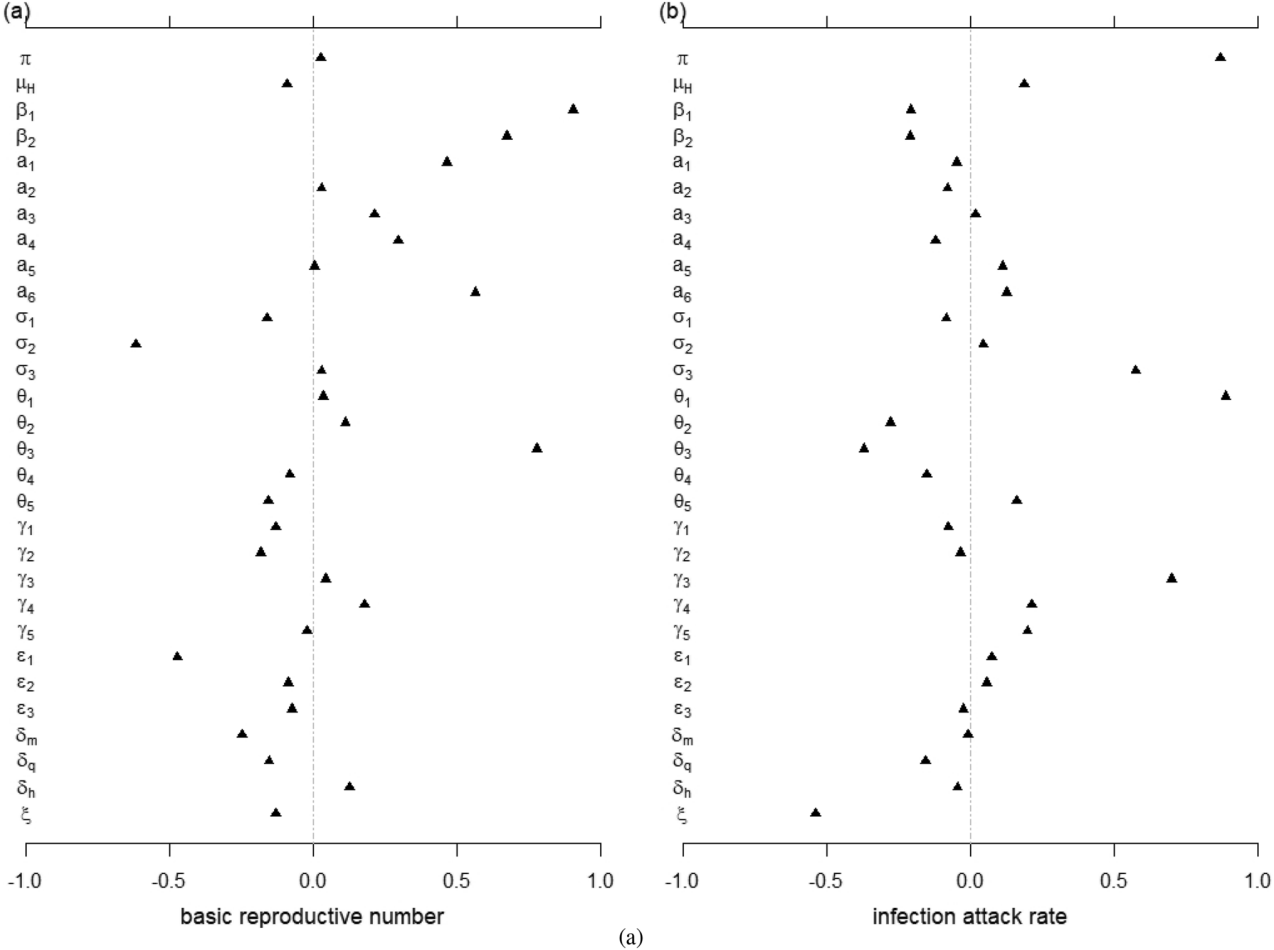
The partial rank correlation coefficient (PRCC) of the basic reproduction number ${\mathscr {R}}_{0}$ and infection attack rate with respect to model parameters.

**Figure 3:**
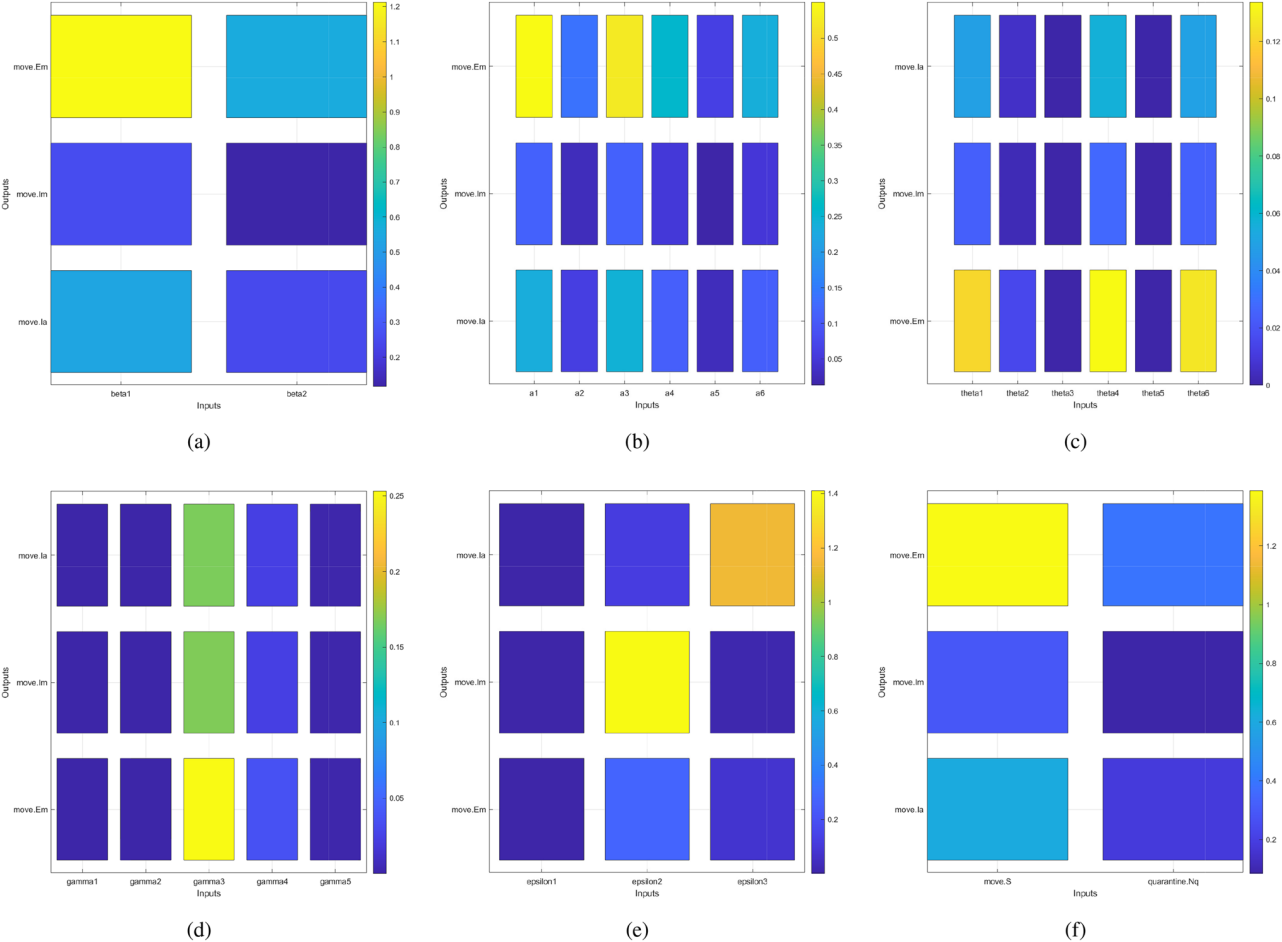
Global sensitivity analysis. Inputs: (**a**) transmission rate ($\beta _{1}$ and $\beta _{2}$), (**b**) effective contact ratio ($a_{1}$–$a_{6}$), (**c**) transition rate [$\theta _{1}$–$\theta _{5}$ and $\theta _{6}=(1-\theta _{2}-\theta _{3}$)], (**d**) recovery rate ($\gamma _{1}$–$\gamma _{5}$), (**e**) hospitalized rate ($\epsilon _{1}$-$\epsilon _{3}$), and (**f**) population size (*S* and $N_{q}$). Output: exposed individuals with outside movement ($E_{m}$), symptomatic infectious individuals ($I_{m}$) and asymptomatic infectious individuals ($I_{a}$).

Sensitivity analysis was conducted to explore the impacts of the mutations in infectiousness, gathering restrictions, and quarantine policies on the dynamical system described in the model (1). The basic reproduction number ${\mathscr {R}}_{0}$, also known as a threshold quantity, is used to assess whether the disease can spread or will die out, though it is not the only factor. Meanwhile, the severity of an outbreak is reflected by the infection attack rate^25^. This paper used Partial Rank Correlation Coefficients (PRCCs)^26^ to investigate the impacts of each parameter on the overall dynamics, with ${\mathscr {R}}_{0}$ and the infection attack rate as response functions (see Fig. 2). Furthermore, using the tool SimBiology in MATLAB^27^, this paper adopted a global sensitivity analysis (shown in Fig. 3) between parameters and variables related to the force of infection (i.e., $E_m$, $I_a$ and $I_m$).

In Fig. 2, the outputs include the basic reproduction number ${\mathscr {R}}_{0}$ (i.e., an epidemiologically key parameter for determining whether the disease will persist) and the infection attack rate (i.e., the severity of an outbreak). The results of the analysis show that four parameters are most significant in their sensitivity:$\beta _1$, $\beta _2$, $\theta _3$ and $a_6$. The transmission rate among susceptible people $\beta _1$ and transfer rate $\theta _3$ from quarantined people $N_q$ to exposed individuals with outside movement $E_m$ ranked as the most sensitive parameters. The transmission rate $\beta _2$ among quarantined people, which significant, is less sensitive than that among susceptible people (i.e., $\beta _1$). The effective contact ratio $a_6$ between asymptomatic infectious individuals $I_a$ and quarantined individuals $N_q$ is the most sensitive parameter among all effective contact ratios. The four significant parameters should especially be taken into consideration by decision-makers in designing and enacting measures for timely and effective infection control.

In Fig. 3, transmission rate $\beta _{1}$ is more sensitive to three outputs (i.e., $E_m$, $I_m$ and $I_a$) than $\beta _{2}$. When any mutated variant attacks susceptible people in the absence of restrictions on movement, its transmission risk will be almost double that of quarantined people. Exposed individuals $E_m$ is the most sensitive group among all infectors. Effective contact ratio $a_{1}$ between $E_m$ and *S* ranked as having the most significant effect on the outputs. Meanwhile, all effective contact ratios ($a_{1},..., a_{6}$) have a greater impact on $E_m$ compared to the other two outputs $I_m$ and $I_a$. In addition, $E_m$ is also affected by transfer rates between disease status compartments (i.e., $\theta _{1}$, $\theta _{4}$, $\theta _{6}=1-\theta _{2}-\theta _{3}$), thus $E_m$ implicitly indicates the effectiveness of quarantine policies. Recovery rate $\gamma _{4}$ of hospitalized symptomatic individuals $H_s$ shows significant impacts on all outputs, especially $E_m$. The hospitalization rates of $I_q$ (i.e., $\epsilon _{2}$) and $I_a$ (i.e., $\epsilon _{3}$) both show an obvious sensitivity to themselves (i.e., $I_q$ or $I_a$) respectively. As shown in Fig. 3(f), the sharp increase in the infected population might be triggered by the influx of inbound travellers who do not quarantine. A quarantine policy for cross-boundary travellers is still suggested.

### Fitting results

**Figure 4:**
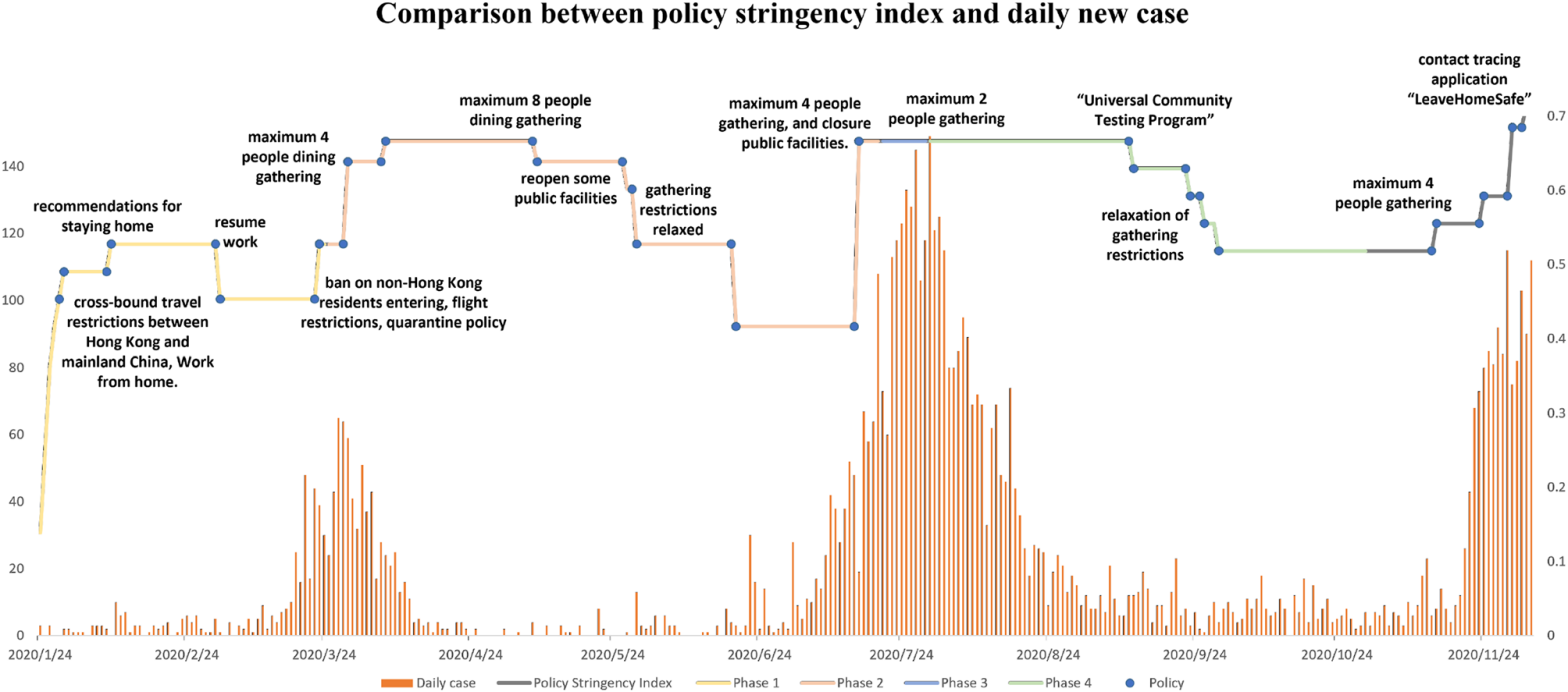
Comparison between policy stringency index scores^16^ and daily new cases^17^ from 24th January to 4th December 2020. Yellow, orange, blue and green lines represent the periods from Jan. 24 to Mar. 24, from May 25 to July 19, from July 20 to 29, and from July 30 to Oct. 30 respectively. Blue points show each inflection point of the grey line “policy stringency index”.

**Figure 5:**
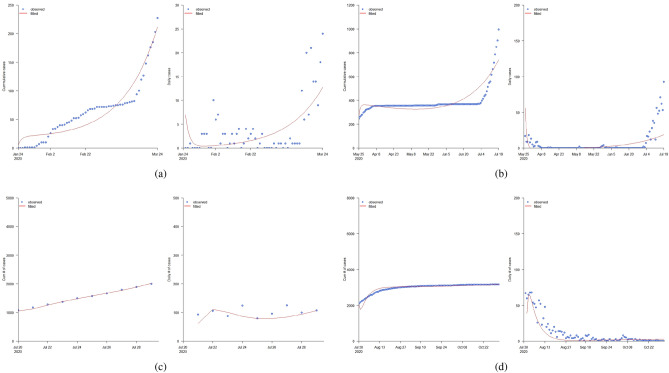
Fitting results: (**a**) Jan. 24 to Mar. 24, (**b**) Mar. 25 to Jul. 19, (**c**) Jul. 20 to Jul. 29, and (**d**) Jul. 30 to Oct. 31.

This study used time-series data on confirmed COVID-19 cases^17^ and data on cross-boundary travellers^28^ in Hong Kong to populate the proposed model. Table 2 provides the descriptions, the initial values, and ranges for each parameter according to reasonable assumptions and previous studies^8,29–31^. Demographic information on the studied population is shown in SI.1. Policy stringency index scores^16^, specific policies implemented at each inflexion point, and the number of new daily cases from 24th January to 4th December 2020 are compared in Fig. 4. The full equations in the model (1) are fitted to symptomatic cases with outside movement $I_{m}$ as shown in Fig. 5. The “R-squared” $R^2$ ranges from 0.69 to 0.98, specifically 0.82 (Phase 1: 24th Jan.–24th Mar.), 0.96 (Phase 2: 25th May.–19th Jul.), 0.98 (Phase 3: 20th Jul.–29th Jul.) and 0.69 (Phase 4: 30th Jul.–31st Oct.) respectively. Model simulations well fitted both the cumulative and daily data. After the quarantine policy was announced on 24th March 2020, the government relaxed, then tightened, then once again relaxed gathering restrictions on 29th May, 19th July and 11st September 2020, respectively. This study separated the study period (282 days) into four subsections because the pandemic in Hong Kong occurred in four waves. At the same time, the government adjusted gathering restrictions or/and quarantine rules every time the daily confirmed cases increased significantly, as shown in Fig. 4. All data can be found on GitHub^32^ and all initial values of variables and estimated parameters (i.e., $a_1$–$a_5$, $\beta _1$, $beta_2$ and $\theta _3$) are shown in SI.4.

**Table 2:** Parameter descriptions and values.

Parameter	Description	Initial value	Range	Citation
$\pi$	The number of new natural births	225	100, 1000	^29^
$\mu _{H}$	The number of inbound travellers without quarantine	0.00003	0.00001, 0.00005	^29^
$\beta _{1}$	Transmission rate contributed by the disease among *S*	0.745	0.36, 1.2	^8^
$\beta _{2}$	Transmission rate contributed by the disease among $N_{q}$	0.745	0.54, 1.7	^8^
$a_{1}$	The effective contact ratio between $E_{m}$ and *S*	0.18	0.11, 0.18	Estimated from^30,31^
$a_{2}$	The effective contact ratio between $I_{a}$ and *S*	0.12	0.05, 0.17	Estimated from^30,31^
$a_{3}$	The effective contact ratio between $I_{m}$ and *S*	0.15	0.1, 0.19	Estimated from^30,31^
$a_{4}$	The effective contact ratio between $E_{m}$ and $N_{q}$	0.13	0.05, 0.18	Estimated from^30,31^
$a_{5}$	The effective contact ratio between $I_{a}$ and $N_{q}$	0.09	0.09, 0.16	Estimated from^30,31^
$a_{6}$	The effective contact ratio between $I_{m}$ and $N_{q}$	0	0, 1	Assumed
$\theta _{1}$	The rate of susceptible individuals who self-quarantined according to the strict policy	0.069	0.01, 0.18	Validated
$\theta _{2}$	The rate of quarantined individuals who remain susceptible after 14-day quarantine observation period and return back to the susceptible group	0.084	0.002, 0.1	Validated
$\theta _{3}$	The rate of quarantined individuals who have been infected during the quarantine period and show the symptoms after the quarantine	0.44	0.075, 0.5	Validated
$\theta _{4}$	The rate of exposed individual with outside movement who has been quarantined	0.084	0.002, 0.1	Validated
$\theta _{5}$	The rate of infectious individual with outside movement who has been quarantined	0.09	0.001, 0.3	Validated
$\sigma _{1}$	The transition rate from exposed to asymptomatic infectious status	0.025	0.001, 0.07	Estimated from^30^
$\sigma _{2}$	The transition rate from exposed to symptomatic infectious status	0.187	0.1, 0.255	Validated
$\sigma _{3}$	The transition rate from exposed to symptomatic infectious status under quarantine	0.289	0.1, 0.3	Validated
$\epsilon _{1}$	The hospitalization rate of asymptomatic infectious individuals	0.8	0.025, 0.95	Assumed
$\epsilon _{2}$	The hospitalization rate of symptomatic infectious individuals	0.85	0.05, 0.975	Assumed
$\epsilon _{3}$	The hospitalization rate of quarantined symptomatic infectious individuals	0.96	0.02, 0.99	Assumed
$\gamma _{1}$	The rate of asymptomatic infectious individuals who recovered without hospitalization	0.008	0.01, 0.45	Assumed
$\gamma _{2}$	The rate of symptomatic infectious individuals who recovered without hospitalization	0.133	0.0714, 0.3333	^30^
$\gamma _{3}$	The rate of quarantined symptomatic infectious individuals who recovered without hospitalization	0.134	0.0714, 0.3333	^30^
$\gamma _{4}$	The rate of symptomatic infectious individuals who recovered after treatment in the hospital	0.116	0.0714, 0.3333	Validated
$\gamma _{5}$	The rate of asymptomatic infectious individuals who recovered after treatment in the hospital	0.005	0.001, 0.5	Assumed
$\delta _{m}$	The rate of death among symptomatic infectious individuals with outside movement $I_{m}$	0.1275	0.01, 0.345	Assumed
$\delta _{q}$	The rate of death among quarantined symptomatic infectious individuals $I_{q}$	0.1275	0.01, 0.345	Assumed
$\delta _{h}$	The rate of death among hospitalized symptomatic infectious individuals $H_{s}$	0.1275	0.01, 0.345	Assumed
$\xi$	The rate of reinfection based on no lifelong immunity	0.0001	0, 1	Assumed

#### Effective contact ratio

Individuals are highly likely to get infected by other pre-symptomatic, asymptomatic or symptomatic individuals through effective contact. Some large-scale studies indicate that greater human mobility may lead to higher infection probability^8,33^, but these studies have failed to assess the influence of this mobility on a heterogeneous population from both epidemiology and policy perspectives. This study explored the effective contact between different groups based on the proposed model.

**Figure 6:**
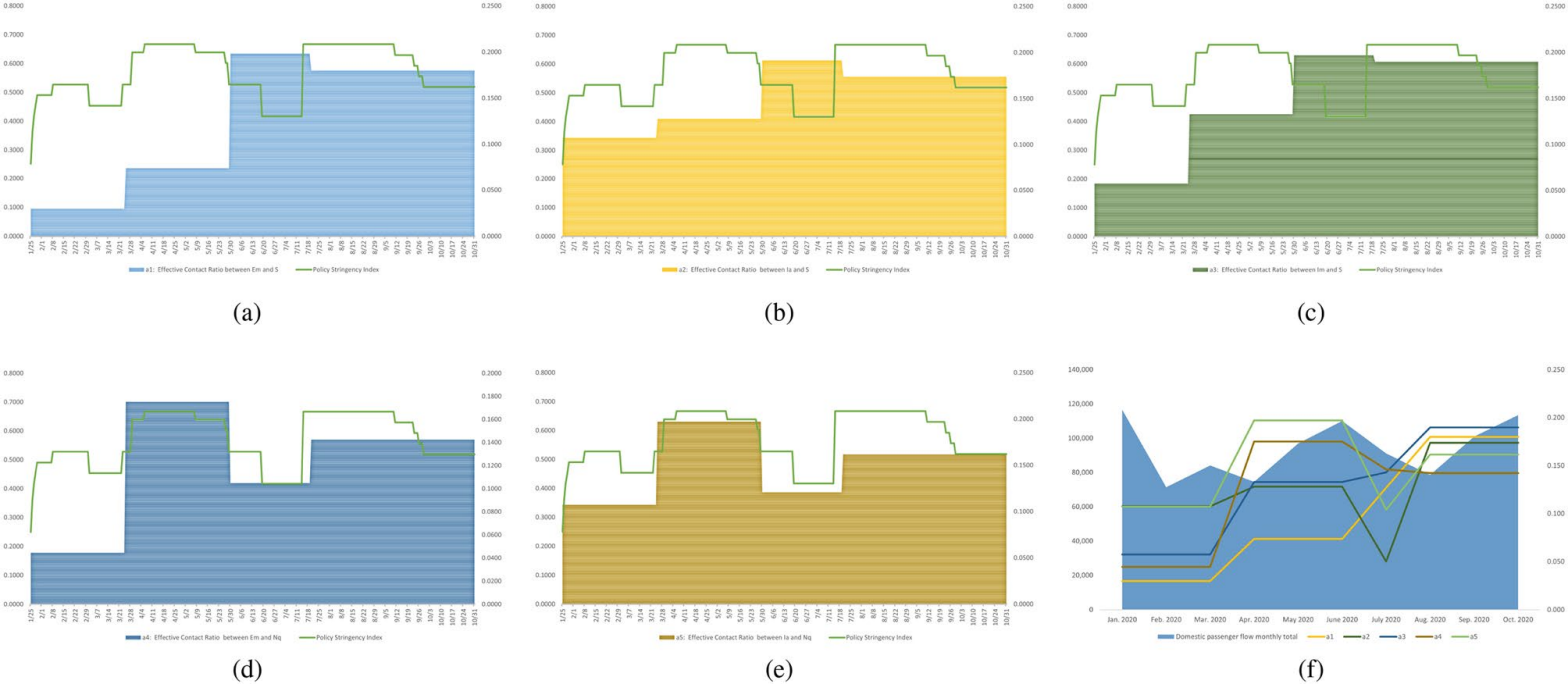
Comparison between policy stringency index scores and effective contact ratios from Jan. 25 to Oct. 31 in 2020: (**a**) $a_{1}$ effective contact ratio between $E_m$ and *S*, (**b**) $a_{2}$ effective contact ratio between $I_m$ and *S*, (**c**) $a_{3}$ effective contact ratio between $I_a$ and *S*, (**d**) $a_{4}$ effective contact ratio between $E_m$ and $N_q$, (**e**) $a_{5}$ effective contact ratio between $I_m$ and $N_q$ and (**f**) comparison between domestic Mass Transit Railway (MTR) passengers flow and effective contact ratios^34^.

In Fig. 6, all effective contact ratios (i.e., $a_{1}-a_{5}$) were at the lowest level before March 2020. After quarantine rules were announced in March 2020, the daily number of cross-boundary travellers gradually decreased from 228 to 1 on average^34^. Two effective contact ratios (i.e., $a_{4}$ and $a_{5}$) related to quarantined people did not decrease, suggesting that quarantined people may become infected during the quarantine. Meanwhile, when the transfer rate $\theta _{3}$ from $N_q$ to $E_m$ grew, the severity of infections during the quarantine became more serious. Owing to the relaxation of gathering restrictions during the gap between the second and third wave, $a_{1}$, $a_{2}$ and $a_{3}$ increased by 0.1245, 0.0632, and 0.0639, respectively. $a_{4}$ and $a_{5}$ decreased by 0.0703 and 0.0763 due to tighter quarantine restrictions. When the third wave came and peaked in mid-July, a restaurant dine-in ban after 6 pm did not curb gatherings among citizens or mitigate the ongoing wave. One week later on 29th July, a stricter gathering restriction was announced and decreased $a_{1}$, $a_{2}$ and $a_{3}$ by 8% on average. Meanwhile, $a_{4}$ and $a_{5}$ increased by 25% in total. Compared to the effective contact ratios in March, all effective contact ratios had a twofold increase even with the implementation of stricter measures, and domestic passenger flows increased as well in late July 2020.

## Discussion

The modified SEIHR model (1) describes the transmission dynamics of SARS-CoV-2 by incorporating heterogeneous effective contact ratios between different groups. Via mathematical analysis, we computed the basic reproduction number, ${\mathscr {R}}_{0}$ (which determines whether the disease persists or dies out) and stability of equilibria. We find that the model exhibits the phenomenon of backward bifurcation, which increases the difficulty of SARS-CoV-2 control since the dynamics do not depend solely on ${\mathscr {R}}_{0}$. The existence of a BB means that when a stable endemic equilibrium co-exists with a stable disease-free equilibrium, even if the basic reproduction number is less than unity, the disease may persist. The epidemiological consequence of the backward bifurcation phenomenon makes the controlling or eliminating the disease more difficult. Potential epidemiological mechanisms of continued transmission may include exogenous re-infection as frequently observed for COVID-19 and imperfect vaccine efficacy due to virus mutations^35^. These and other possible mechanisms require further study.

The main impact factors of ${\mathscr {R}}_{0}$ shown in Eq.(5) are effective contact ratios $a_{1},a_{2}$ and $a_{3}$ controlled by gathering restrictions, and effectiveness contact ratios between quarantined inbound travellers and infectious individuals outside (i.e., $a_{4}$ and $a_{5}$) and the transition rate $\theta _{3}$ from $N_{q}$ to $I_{m}$ which reflect potential infection during quarantine. This study assumed the effective contact ratio $a_6$ between $I_m$ and $N_q$ to be zero since people visiting $N_q$ are assumed to be without symptoms. In Fig. 2, $a_6$ showed high sensitivity to ${\mathscr {R}}_{0}$ and the infection attack rate, a result that indicates the contact between $I_m$ and $N_q$ should be emphasised for overall control. As for quarantined individuals, incomplete adherence to quarantine recommendations could potentially accelerate and prolong infectious disease outbreaks. Transition rate $\theta _{3}$ was validated to be greater than zero, which confirms the existence of infections during quarantine. There are at least two transmission links. Some inbound travellers are susceptible before being quarantined and get infected by their close contacts. In addition, inbound travellers still have the possibility of infecting their close contacts due to the high frequency of secondary infections from imported cases^17^. Given the lesson from Australia^36^, if any infection is passed from quarantined individuals to their close contacts, the virus may spread into the community, resulting in an outbreak.

Additional evidence of infections possibly occurring during quarantine includes the increases in $a_{4}$ and $a_{5}$ in late March and late July 2020. A higher effective contact ratio increases the possibility of shedding the virus in the population and infecting others. Up until the end of July 2020, the policy stringency index score is almost 2 times higher than that in late January 2020^16^. Contrary to the expected change shown in Fig. 6, even the best performance, i.e., the effective contact ratio $a_{1}$ between $E_{m}$ and *S*, increased by 0.15 in total. Owing to no symptoms and delayed recognition by decision makers of the relative transmissibility of asymptomatic infection, effective contact ratios related to $I_{a}$ indicate low adherence to gathering restrictions, exhibiting the second highest increase of all the effective contact ratios between January and October 2020.

After implementing a stricter restriction, $a_{1}$, $a_{2}$ and $a_{3}$ decreased only 8% on average while the third wave reached its peak. After gathering restrictions were relaxed in September 2020 to allow a maximum of four people together, the effective contact ratios, with an average increase of 0.0475, were approximately six times greater than that in July 2020. The synchronized effective contact ratio didn’t change with the stringency of gathering restrictions. This implies that people practiced lower and lower adherence to policies. Hong Kong may have experienced pandemic fatigue in their populations in July 2020, with the most severe resurgence occurring in September 2020.

The occurrence of backward bifurcation, infection during quarantine, and pandemic fatigue may be reasons why Hong Kong experienced multiple waves of infection during the COVID-19 pandemic. Pandemic fatigue was expressed through an increasing number of people not sufficiently following recommendations and restrictions, as reflected in effective contact ratios. Already infected individuals who volunteer to provide daily necessities to quarantined individuals may, despite the very quarantine policy, infect the quarantined individuals who, after quarantine and when initially asymptomatic, interact with and infect other community members, triggering an outbreak. According to guidance from WHO^4^, we need to apply more tailored measures to allow people to live their lives but reduce risks.

In February 2021, the Hong Kong government announced sewage tests for COVID-19 and promoted the “LeaveHomeSafe mobile application to record citizens’ social activities. With these measures in place, the government also implemented a policy such that if one or more new confirmed cases with unknown sources are found in buildings, or there are sewage samples that test positive and thus imply possible infection risks, the buildings will be included in a mandatory test notice^37^. The sewage tests detected nine infections in two blocks^38^. Using data collected through the “LeaveHomeSafe” application, the Hong Kong government was able to efficiently identify close contacts traceable to an infection cluster that occurred in the K11 Musea shopping center^39^. More cross-cutting measures and their efficacy need to be explored. Finally, this study can be extended by examining the time-varying effective contact ratios using more detailed data, incorporating heterogeneous data to gain further insight on the contacts between different groups, and exploring more tailored policies and their efficacy.

## Methods

### Mathematical model

#### Model formulation

The epidemic model used in this study follows the compartment model from Kermack and McKendrick^5^. COVID-19 has a wide range of the targeted susceptible group and various complications (e.g., fever and cough)^40^. Considering implemented anti-epidemic strategies, the diagram is shown as Fig. 1. The modified SEIHR model is presented in ordinary differential equations (1). All variables and parameters are described in Tables 2 and 3. We split the total human population at time *t* , denoted by susceptible individuals *S*(*t*), quarantined inbound travellers $N_{q}(t)$, exposed individuals with outside movement $E_{m}(t)$, quarantined exposed individuals $E_{q}(t)$, asymptomatic infectious individuals $I_{a}(t)$, symptomatic infectious individuals with outside movement $I_{m}(t)$, quarantined symptomatic infectious individuals $I_{q}(t)$, hospitalized asymptomatic infectious individuals $H_{a}(t)$, hospitalized symptomatic infectious individuals $H_{s}(t)$ and recovered individuals *R*(*t*).

The labels *q*, *m*, *a* and *s* represent “quarantined”, “with movement”, “asymptomatic” and “symptomatic” respectively. Given that the life expectancy in Hong Kong is 84.89, this study considers the daily natural birth $\pi$ as “225” and the natural death rate $\mu _{{H}}$ as “0.00003”^29^. Rare reinfections of COVID-19 caused by various viral isolates have been reported^41^. We assumed the reinfected probability to be a constant parameter $\xi$ with a value of “0.0001”. $\delta _{k}\,(k = m, q, h)$, is the death rate among $I_{m}$, $I_{q}$ and $H_{s}$. In Fig. 1, all arrows are labelled with the transition rates between compartments. $\theta _{k}$ $(k=1,2,3,4,5)$, is the percentage rate of a given population in one compartment transferring to another compartment. A positive term ($1-\theta _{2}-\theta _{3}$) represents the probability of a quarantined inbound traveller being infected before the quarantine. $\frac{1}{\sigma _{k}}$ $(k=1,2,3)$ is the average duration of the latency period, i.e., the time between when an individual is exposed to the virus and when the individual starts to infect others. $\epsilon _{k}$ $(k=1,2,3)$ represents the hospitalization rate and $\gamma _{k}$ $(k=1,2,3,4,5)$ is the mortality among each group $I_{k}$ $(k=a,m,q)$ or $H_{k}$ $(k=a,s)$. Each parameter ranges from 0 to 1. The force of infection $\lambda _{1}$ and $\lambda _{2}$ contain the transmission rates $\beta _{1}$ and $\beta _{2}$ due to the characteristics of the disease itself and due to interactions between members of the population as indicated through the effective contact ratio $a_{i}$ $(i=1,2,3,4,5,6)$. The initial population equals the number of local residents at the end of 2019: 7,520,800^42^.

1 $$\begin{aligned} \left\{ \begin{array}{l} \frac{dS}{dt}=\pi + m_{N} +\xi \,R+\theta _{{2}}\,N_{{q}}-(\theta _{{1}}+\mu _{{H}}+\lambda _{1})\,S , \\ \frac{dN_{q}}{dt}=m_{N_{q}}+\theta _{{1}}\,S-(\theta _{{2}}+\mu _{{H}}+(1-\theta _{2}-\theta _{3})+\theta _{3}\lambda _{2})\,N_{q}, \\ \frac{dE_{{m}}}{dt}=\lambda _{1}\,S+\theta _{3}\lambda _{2}\,N_{q}-(\sigma _{{1}}+\sigma _{{2}}+\theta _{{4}}+\mu _{{H}})\,E_{{m}} , \\ \frac{dE_{{q}}}{dt}=(1-\theta _{2}-\theta _{3})\,N_{q}+\theta _{{4}}\,E_{{m}}-(\sigma _{{3}}+\mu _{{H}})\,E_{{q}} ,\\ \frac{dI_{{a}}}{dt}=\sigma _{{1}}\,E_{{m}}-(\gamma _{{1}}+\epsilon _{{3}}+\mu _{{H}})I_{{a}} ,\\ \frac{dI_{{m}}}{dt}=\sigma _{{2}}\,E_{{m}}-(\gamma _{{2}}+\epsilon _{{1}}+\delta _{{m}}+\theta _{{5}}+\mu _{{H}})\,I_{{m}} ,\\ \frac{dI_{{q}}}{dt}=\sigma _{{3}}\,E_{{q}}+\theta _{{5}}\,I_{{m}}-(\gamma _{{3}}+\epsilon _{{2}}+\delta _{{q}}+\mu _{{H}})\,I_{{q}} ,\\ \frac{dH_{{a}}}{dt}=\varepsilon _{{3}}\,I_{{a}}-(\gamma _{{5}}+\mu _{{H}})\,H_{{a}} ,\\ \frac{dH_{{s}}}{dt}=\varepsilon _{{1}}\,I_{{m}}+\varepsilon _{{2}}\,I_{{q}}-(\gamma _{{4}}+\delta _{{h}}+\mu _{{H}})\,H_{{s}} ,\\ \frac{dR}{dt}=\gamma _{{1}}I_{{a}}+\gamma _{{2}}I_{{m}}+\gamma _{{3}}I_{{q}}+\gamma _{{4}}H_{{s}}+\gamma _{{5}}H_{{a}}-(\xi +\mu _{{H}})\,R \end{array} \right. \end{aligned}$$

where the force of infection is given by:

2 $$\begin{aligned} \lambda _{1}={\frac{\beta _{1}(a_{{1}}E_{{m}}+a_{{2}}I_{{a}}+a_{{3}}I_{{m}})}{N}}, \,\, \lambda _{2}={\frac{\beta _{2}(a_{{4}}E_{{m}}+a_{{5}}I_{{a}}+a_{{6}}I_{{m}})}{N}}, \end{aligned}$$

with *N* representing the total population at time *t* given by $N(t)=S(t)+N_{{q}}(t)+E_{{m}}(t)+E_{{q}}(t)+I_{{a}}(t)+I_{{m}}(t)+I_{{q}}(t)+H_{{a}}(t)+H_{{s}}(t)+R(t)$.

**Table 3:** Notation.

Notation	Description
**Variables**	
*S*	The number of susceptible individuals
$N_{{q}}$	The number of quarantined inbound travellers
$E_{{m}}$	The number of exposed individuals with outside movement
$E_{{q}}$	The number of quarantined exposed individuals
$I_{{a}}$	The number of asymptomatic infectious individuals
$I_{{m}}$	The number of symptomatic infectious individuals with outside movement
$I_{{q}}$	The number of quarantined symptomatic infectious individuals
$H_{{a}}$	The number of hospitalized asymptomatic infectious individuals
$H_{{s}}$	The number of hospitalized symptomatic infectious individuals
*R*	The number of recovered individuals
**Parameters**	
$m_{{N}}$	The number of inbound travellers without quarantine
$m_{{N_{{q}}}}$	The number of quarantined inbound travellers

The force of infection $\lambda _{1}$ and $\lambda _{2}$ are driven by $E_{m}$, $I_{a}$ and $I_{m}$. As for the quarantined group, $N_q$ may transition to one of three groups, including back to susceptible group *S*, with symptom onset during quarantine $E_q$ or after quarantine as $E_m$. We assume that if a quarantined individual is infected during the quarantine period, symptoms would appear after the quarantine. In addition, the government will ask the individual’s close contacts to comply with a 14-day compulsory quarantine. $E_q$ is made up of individuals who were exposed to the virus through contact with quarantined inbound travellers or other close contacts. The specific moment when an exposed individual becomes exposed and pre-symptomatic is unknown. In this study, we assume the average latency period is three days. All exposed individuals are also assumed to be pre-symptomatic and can transmit the virus, and all quarantined infectious individuals are assumed to be symptomatic. Owing to their obvious symptoms, $I_m$ cannot be regarded as a related individual who can take care of the quarantined people: $a_6 = 0$.

### Mathematical analysis

In this section, a brief summary of the mathematical analysis underlying this study is provided. The equations in the model (1) are defined as a positive dynamical system with the domain $\Omega$. The stability of equilibria is formulated in terms of the next generation method^24^ and bifurcation theory^43^. Firstly, we consider solutions to (1), which is given by

$$\begin{aligned} \Omega = \{(S,N_{{q}},E_{{m}},E_{{q}},I_{{a}},I_{{m}},I_{{q}},H_{{a}},H_{{s}},R) \in \mathbb {Z}^{10}_+:N > 0 \}. \end{aligned}$$

Thus, simplifying *N* from model (1) i.e., $N^{'}= S^{'}...+R^{'}$, one can clearly see that all solutions to the model that start in $\Omega$ will remain in $\Omega$ for all $t \ge 0$. Hence, $\Omega$ is positive-invariant, and it is sufficient to determine solutions that are restricted to $\Omega$. Therefore, for the model (1), the existence, uniqueness, and continuation results hold provided the solutions that are restricted to $\Omega$ hold^44,45^.

#### Disease-free equilibrium (DFE)

The DFE showed a locally asymptotic stablility with the initial condition^24^: only *S*(0) and $N_{{q}}(0)$ are not equal to zero, and other variables should be equal to zero or much less than *S*(0) and $N_{{q}}(0)$.

$$\begin{aligned} \Omega _{1} = [ S(0),N_{{q}}(0),E_{{m}}(0),E_{{q}}(0),I_{{a}}(0),I_{{m}}(0),I_{{q}}(0),H_{{a}}(0),H_{{s}}(0),R(0) ] = [ S_{{0}},{N_{q}}_{{0}},0,0,0,0,0,0,0,0 ]. \end{aligned}$$

The matrix for the new infection terms is designated as *F*. The matrices of the remaining individuals transferring out of (into) compartments are represented as $V^{-}$ and $V^{+}$. The transition term *V* is the difference between $V^{-}$ and $V^{+}$. Based on the equations in model (1),the DFE of *S* is as follows:

3 $$\begin{aligned} \begin{aligned} S_{{0}}^{*}= {\frac{(\pi +m_{{N}}) \mu _{{H}}+ (\pi +m_{{N}})(1-\theta _{{3}})+m_{{N_{{q}}}}\theta _{{2}}}{{\mu _{{H}}}^{2}+ (1+ \theta _{{1}}-\theta _{{3}})\mu _{{H}}+\theta _{{ 1}}(1-\theta _{{2}}-\theta _{{3}})}}. \end{aligned} \end{aligned}$$

Substituting Eq. (3) into (1),we obtain

4 $$\begin{aligned} \begin{aligned} {N_{q}}_{{0}}^{*}={\frac{(\pi +m_{{N}}+m_{{N_{{q}}}} )\theta _{{1}}+m_{{N_{{q}}}}\mu _{{H}}}{(1+ \mu _{{H}}-\theta _{{2}}-\theta _{{3}})\theta _{{1}}+\mu _{{H}}(\mu _{{H}}+1-\theta _{{3}}) }}. \end{aligned} \end{aligned}$$

where   $$0<\theta _{2}+\theta _{3} < 1$$   and  $$0< \theta _{1} < 1$$.

Hence, $S_{{0}}^{*}$ and ${N_{q}}_{{0}}^{*}$ are both positive.

Applying the next-generation method^24^ to the equations in the model (1), which is described in SI.2, the basic reproduction number, ${\mathscr {R}}_{0}$, is given by

5 $$\begin{aligned} \begin{aligned} {\mathscr {R}}_{0}= \rho {(F{V}^{-1})}= \frac{(a_{4}\beta _{2}\theta _{3}+a_{1}\beta _{{1}})q_{3}q_{4} + (a_{6}\beta _{2}\theta _{3}+a_{3}\beta _{1})\sigma _{2}q_{3} + (a_{5}\beta _{2}\theta _{3}+a_{2}\beta _{1})\,\sigma _{1}q_{4}}{q_{1}q_{3}q_{4}}, \end{aligned} \end{aligned}$$

where

$$\begin{aligned}{} &q = \theta _{{1}}+\mu _{{H}},\quad q_{{0}} = 1-\theta _{{3}}+\mu _{{H}}, \quad q_{{1}} = \sigma _{{1}}+\sigma _{{2}}+\theta _{{4}}+\mu _{{H}},\quad q_{{2}} = \theta _{{3}}+\mu _{{H}}, \\&q_{{3}} = \gamma _{{1}}+\epsilon _{{3}}+\mu _{{H}}, \quad q_{{4}} = \gamma _{{2}}+\epsilon _{{1}}+\delta _{{m}}+\theta _{{5}}+\mu _{{H}}, \quad q_{{5}} = \gamma _{{3}}+\epsilon _{{2}}+\delta _{{q}}+\mu _{{H}}, \\&q_{{6}} = \gamma _{{5}}+\mu _{{H}}, \quad q_{{7}} = \gamma _{{4}}+\delta _{{h}}+\mu _{{H}} \quad \text {and} \quad q_{{8}} = \xi +\mu _{{H}}. \end{aligned}$$

##### Theorem 1

*The DFE in the model (*1*) is locally-asymptotically stable when* ${\mathscr {R}}_{0}< 1$*, and unstable when* ${\mathscr {R}}_{0}> 1$.

##### *Proof*

The proof of Theorem 1 can be deducted following^24^. $\square$

The basic reproduction number (${\mathscr {R}}_{0}$) represents the average number of secondary infections caused by a single infection when a population is wholly susceptible^24^. The interpretation of ${\mathscr {R}}_{0}$ is shown in Table 1. Based on the initial values estimated from^7,8,30^, the basic reproduction number ${\mathscr {R}}_{0}$ is larger than one. In addition, $E_{m}$ contributed the most infections, which exceeded 80%.

#### Endemic equilibrium (EE)

In this subsection, for mathematical convenience, we assumed that $\lambda _{1}$ and $\lambda _{2}$ are the same:

6 $$\begin{aligned} \begin{aligned} \lambda _{1}=\lambda _{2}=\lambda =\frac{\beta (a_{{1}}E_{{m}}+a_{{2}}I_{{a}}+a_{{3}}I_{{m}})}{N}. \end{aligned} \end{aligned}$$

The EE is a scenario where a disease persists in a population. The globally asymptotic stability of the EE exists when ${\mathscr {R}}_{0}> 1$ and the infected compartments are non-empty. Suppose $\Omega _{2}$ is given as

$$\begin{aligned} \Omega _{2} = [S^*,N_{{q}}^*,E_{{m}}^*,E_{{q}}^*,I_{{a}}^*,I_{{m}}^*,I_{{q}}^*,H_{{a}}^*,H_{{s}}^*,R^*]. \end{aligned}$$

Given the Eq. (1), we obtain

7 $$\begin{aligned}&E_{q}^*={\frac{ ( \theta _{{4}}-( h_{{1}}q_{{6}}+h_{ {2}} )\xi \,t_{{2}}{\omega }^{-1} ) E_{{m}}^*-(\xi \,t_{{4}}t_{{3}} \theta _{{1}}+m_{{N_{{q}}}}q_{{0}}t_{{4}} )t_{{2}}{\omega }^{-1}}{q_{{2}}}}, \end{aligned}$$

8 $$\begin{aligned}&I_{a}^*={\frac{\sigma _{{1}}E_{{m}}^*}{q_{{3}}}}, \end{aligned}$$

9 $$\begin{aligned}&I_{m}^*={\frac{\sigma _{{2}}E_{{m}}^*}{q_{{4}}}}, \end{aligned}$$

10 $$\begin{aligned}&I_{q}^*={\frac{ ( q_{{2}}\sigma _{{2}}\theta _{{5}}+\theta _{{4}}t_{{2}}{\omega }^{-1}\,\sigma _{{3}}q_{{4}}-\xi \,t_{{2}}{\omega }^{-1}\,\sigma _{{3}}q_{{4}}( h_{{1}}q_{{6}}+h_{{2}} ) ) E_{{m}}^*+ ( t_{{3}}\theta _{{1}}\xi +m_{{N_{{q}}}}q_{{0}} ) t_{{2}}t_{{4}}\sigma _{{3}}q_{{4}}{\omega }^{-1}}{q_{{2}}q_{{4}}q_{{5}}}}, \end{aligned}$$

11 $$\begin{aligned} H_{a}^*={\frac{\epsilon _{{3}}\sigma _{{1}}E_{{m}}^*}{q_{{3}}q_{{6}}}} , \end{aligned}$$

12 $$\begin{aligned}&H_{s}^*={\frac{ ( \sigma _{{2}}\epsilon _{{1}}q_{{2}}q_{{5}}+\sigma _{{2}} \theta _{{5}}q_{{2}}\epsilon _{{2}}- (( h_{{1}}q_{{6}}+h_{{2}} ) \xi -\theta _{{4}} )\sigma _{{3}}t_{{2}}q_{{4}}{\omega }^{-1}) E_{{m}}^*-\epsilon _{{2}}t_{{4}}q_{{4}} ( \xi \,t_{ {3}}\theta _{{1}}+m_{{N_{{q}}}}q_{{0}} ) }{q_{{2}}q_{{4}}q_{{5}}q _{{7}}}}, \end{aligned}$$

where

$$\begin{aligned} \begin{aligned} t_{1}=&\lambda \,\theta _{{3}}+q, \qquad \qquad \qquad \qquad t_{2}=\theta _{{2}}+\theta _{{3}}-1, \qquad \qquad \qquad \quad t_{3}=\pi +m_{{N}}\,,\\ t_{4}=&q_{{8}}q_{{4}}q_{{6}}q_{{7}}q_{{3}}q_{{2}}q_{{5}}, \qquad \qquad \qquad t_{5}=\gamma _{{2}}q_{{5}}+\gamma _{{3}}\theta _{{5}}, \qquad \qquad \qquad t_{6}=q_{{5}}\epsilon _{{1}}+\theta _{{5}}\epsilon _{{2}}\,,\\ t_{7}=&-\theta _{{1}}\theta _{{2}}+q_{{0}} ( \lambda \,\theta _{{3}}+q) , \qquad t_{8}=\epsilon _{{2}}\gamma _{{4}}+\gamma _{{3}}q_{{7}}, \qquad t_{9}=( \gamma _{{2}}q_{{5}}+\gamma _{{3}}\theta _{{5}} ) q_{{7}}+\gamma _{{4}} (q_{{5}}\epsilon _{{1}}+\theta _{{5}}\epsilon _{{2}}),\\ \qquad \omega =&q_{{4}} ( ( \xi \, ( \theta _{{2}}+\theta _{{3}}-1 ) ( \epsilon _{{2}}\gamma _{{4}}+\gamma _{{3}}q_{{7}} ) \sigma _{{3}}-q_{{7}}q_{{8}}\theta _{{2}}q_{{2}}q_{{5}} ) \theta _{{1}}+q_{{5}}q_{{7}}q_{{8}}q_{{0}}q_{{2}} ( \lambda \,\theta _{{3}}+q )) q_{6}q_{3}\,,\\ h_{1}=&h_{{11}}+h_{{12}}+h_{{13}}=q_{{7}}\gamma _{{1}}\sigma _{{1}}q_{{2}}q_{{4 }}q_{{5}}+\sigma _{{3}}\theta _{{4}}q_{{3}}q_{{4}}(\epsilon _{{2}}\gamma _{{4}}+\gamma _{{3}}q_{{7}})+(q_{{2}}q_{{3}}\sigma _{{2}} ((\gamma _{{2}}q_{{5}}+\gamma _{{3}}\theta _{{5}}) q_{{7}}+\gamma _{{4}}(q_{{5}}\epsilon _{{1}}+\theta _{{5}}\epsilon _ {{2}}))),\\ h_{2}=&\gamma _{{5}}\sigma _{{1}}\epsilon _{{3}}q_{{2}}q_{{4}}q_{{5}}q_{{7}}, \qquad h_{3}= ( \theta _{{2}}+\theta _{{3}}-1 ) ( \epsilon _{{2}} \gamma _{{4}}+\gamma _{{3}}q_{{7}} ) \sigma _{{3}}q_{{3}}q_{{4}}q_{ {6}} ( m_{{N_{{q}}}}q_{{0}}+ ( \pi +m_{{N}} ) \theta _{{ 1}} ).\\ \end{aligned} \end{aligned}$$

Simplify the model (1) by substituting Eq. (7) to (12), the EE of $S^*$, $N_{q}^*$ and $R^*$ can be rewritten as follows:

13 $$\begin{aligned}&S^*=\frac{( ( h_{{1}}+h_{{2}} ) \xi \,E_{{m}}^*t_{{1}}-h_ {{3}}\xi \,m_{{N_{{q}}}}+t_{{4}} ( m_{{N_{{q}}}}\theta _{{2}}+t_{{1 }}t_{{3}} ) )}{\omega }, \end{aligned}$$

14 $$\begin{aligned}&N_{q}^*=\frac{( ( h_{{1}}q_{{6}}+h_{{2}} ) \xi \,E_{{m}}^*+ \xi \,t_{{4}}t_{{3}}\theta _{{1}}+m_{{N_{{q}}}}q_{{0}}t_{{4}})}{\omega }, \end{aligned}$$

15 $$\begin{aligned}&R^*=\frac{( ( h_{{11}} ( q_{{0}}t_{{1}}-\theta _{{1}} \theta _{{2}} ) + ( h_{{12}}+h_{{13}} ) t_{{7}} ) E_{{m}}^*-h_{{3}})}{\omega }, \end{aligned}$$

and

16 $$\begin{aligned} \begin{aligned} E_{m}^*={\frac{B_{{2}}{\lambda }^{2}+B_{{1}}\lambda }{{\lambda }^{2}A_{{2}}+\lambda \,A_{{1}}+A_{{0}}}}, \end{aligned} \end{aligned}$$

where

$$\begin{aligned} \begin{aligned} B_{1}=&-((( q_{{5}}q_{{8}}(q_{{0}}\theta _{{3}}+\theta _{{2}} ) q_{{2}}-\gamma _{{3}}\sigma _{{3}}\xi \,t_{2}) m_{{N_{{q}}}}+q_{{8}}q_{{2}}q_{{5}}t_{3}(\theta _{{1}}\theta _{{3}}+q))q_{{7}} -\gamma _{{4}}\sigma _{{3}}\epsilon _{{2}}m_{{N_{{q}}}}\xi \,t_{2})q_{{3}}q_{{6}}q_{{4}},\\ B_{2}=&-q_{{6}}q_{{4}}q_{{3}}q_{{2}}q_{{5}}q_{{7}}q_{{8}}\theta _{{3}}t_{3},\\ A_{0}=&-q_{{6}}q_{{4}}q_{{3}}( ( \xi \,t_{2}t_{8}\sigma _{{3}}-q_{{7}}q_{{8}}\theta _{{2}}q_{{2}}q_{{5}})\theta _{{1}}+qq_{{0}}q_{{2}}q_{{5}}q_{{7}}q_{{8}} ) q_{{1}},\\ A_{1}=&( \theta _{{1}}\theta _{{3}}+q ) ( ( ( \sigma _{{2}}t_{9}q_{{3}}+q_{{7}}\gamma _{{1}}\sigma _{{1}}q_{{4}}q_{{5}} ) q_{{2}}+\sigma _{{3}}\theta _{{4}}q_{{3}}q_{{ 4}}t_{8}) q_{{6}}+q_{{7}}\gamma _{{5}}\sigma _{{1}}\epsilon _{{3}}q_{{2}}q_{{4}}q_{{5}} )\xi -q_{{0}}q_{{1}}t_{4}\theta _{{3}},\\ A_{2}=&\theta _{{3}}\xi \, ( ( ( \sigma _{{2}}t_{9}q_{{3}}+q_{{7}}\gamma _{{1}}\sigma _{{1}}q_{{4}}q_{{5}})q_{{2}}+\sigma _{{3}}\theta _{{4}}q_{{3}}q_{{4}}t_{8} )q_{{6}}+q_{{7}}\gamma _{{5}}\sigma _{{1}}\epsilon _{{3}}q_{{2}}q_{{4}}q_{{5}}).\\ \end{aligned} \end{aligned}$$

From above, we express other variables in terms of $E^*$, which is difficult to adjust whether the variables are always positive or not. This study proves the existence of EE in SI.2.

Substituting Eq. (16), (8) and (9) into (6), we obtain

17 $$\begin{aligned} \begin{aligned} \lambda ^*=\frac{\beta (a_{{1}}E_{{m}}^*+a_{{2}}I_{{a}}^*+a_{{3}}I_{{m}}^*)}{N^*}, \end{aligned} \end{aligned}$$

and

18 $$\begin{aligned} \begin{aligned} N^*= S^*+N_{{q}}^*+E_{{m}}^*+E_{{q}}^*+I_{{a}}^*+I_{{m}}^*+I_{{q}}^*+H_{{a}}^*+H_{{s}}^*+R^*.\\ \end{aligned} \end{aligned}$$

Now, substituting the endemic equilibrium points (SI.2) and Eq. (17) into Eq. (18), we have

19 $$\begin{aligned} \begin{aligned} S^*+N_{{q}}^*+(1-\frac{\beta \,a_{1}}{\lambda ^*})E_{{m}}^*+E_{{q}}^*+(1-\frac{\beta \,a_{2}}{\lambda ^*})I_{{a}}^*+(1-\frac{\beta \,a_{3}}{\lambda ^*})I_{{m}}^*+I_{{q}}^*+H_{{a}}^*+H_{{s}}^*+R^*=0. \end{aligned} \end{aligned}$$

Simplifying this equation may point towards the existence of the existence of the backward bifurcation phenomenon, which will be discussed in the subsequent section.

#### Backward bifurcation analysis

When the disease cannot develop into an epidemic, ${\mathscr {R}}_{0}$ is less than unity which is a necessary condition. In considering the possibility of the coexistence of stable DFE and EE, the backward bifurcation (BB) phenomenon is discussed in this section. Here, we simplify the Eq. (5) with “ $\beta _{1}=\beta _{2}$”, “$a_{1}=a_{4}$”, “$a_{2}=a_{5}$” and “$a_{3}=a_{6}$” as follows:

20 $$\begin{aligned} R_0= R_{E_{m}}+R_{I_{a}}+R_{I_{m}}, \end{aligned}$$

with

$$\begin{aligned} {} &R_{E_{m}}=\beta (1+\theta _{3})\,\frac{a_{1}}{q_{3}q_{4}},\\&R_{I_{a}}=\beta (1+\theta _{3})\,\frac{a_{2}\sigma _{1}}{q_{1}q_{3}},\\&R_{I_{m}}=\beta (1+\theta _{3})\,\frac{a_{3}\sigma _{2}}{q_{1}q_{4}}. \end{aligned}$$

Substituting Eq. (17) and (18) into Eq. (19),

21 $$\begin{aligned} \begin{aligned} C_{4}{\lambda ^*}^4+C_{3}{\lambda ^*}^3+C_{2}{\lambda ^*}^2+C_{1}{\lambda ^*}+C_{0}=0, \end{aligned} \end{aligned}$$

where

$$\begin{aligned}&{\begin{aligned} C_{0}=&q_{{3}}( -\epsilon _{{2}}t_{{4}}q_{{4}} ( \xi \,t_{{3}} \theta _{{1}}+m_{{N_{{q}}}}q_{{0}} ) +c_{{0}} ( ( ( ( ( \theta _{{2}}+q_{{0}} ) m_{{N_{{q}}}}+t_{ {3}} ( \xi \,\theta _{{1}}+q ) ) q_{{2}}-t_{{2}} ( \xi \,t_{{3}}\theta _{{1}}+m_{{N_{{q}}}}q_{{0}} ) ) q_{{5}}\\&+ \, t_{{2}}\sigma _{{3}} ( \xi \,t_{{3}}\theta _{{1}}+m _{{N_{{q}}}}q_{{0}} ) ) t_{{4}}-q_{{5}}h_{{3}}q_{{2}} ( \xi \,m_{{N_{{q}}}}+1) ) q_{{4}}q_{{7}} ) q _{{6}}A_{{0}}+ \frac{-B_{1}q_{2}q_{5}q_{6}q_{7}}{1+\theta _{3}}\,({q _{3}}^2{q_{4}}^2R_{E_{m}}+q_{1}q_{3}q_{4}R_{I_{a}}\\&+ \, q_{1}q_{3}q_{4}R_{I_{m}}),\\ \end{aligned}} \\&{\begin{aligned} C_{1}=&(-q_{{3}}q_{{6}}t_{{4}}\epsilon _{{2}}A_{{1}}q_{{4}} ( \xi \,t_{{3} }\theta _{{1}}+m_{{N_{{q}}}}q_{{0}} ) +\xi \,q_{{3}}h_{{1}} ((( q_{{2}}-t_{{2}} ) q_{{5}}-t_{{2}}\sigma _ {{3}} ) q_{{7}}-t_{{2}}\sigma _{{3}} ) B_{{1}}q_{{4}}c_{{0} }{q_{{6}}}^{2}+ ( ( ( ( ( ( ((( qh_{{1}}\\& + \, h_{{2}} ( q+1 ) ) \xi +h_{{1}} (q\,q_{{0}}-\theta _{{1}}\theta _{{2}} ) ) B_{{1}}+ ( \xi \,t_{{3}}\theta _{{1}}A_{{1}}+ ( ( \theta _{{2}}+q_{{0}} ) m_{{N_{{q}}}}+qt_{{3}} ) A_{{1}}+t_{{3}} \theta _{{3}}A_{{0}} ) t_{{4}}-h_{{3}}A_{{1}} ( \xi \,m_{{N_{ {q}}}}\\&+ \, 1 ) ) c_{{0}}+B_{{1}}+ ( c_{{1}}t_{{3}}\theta _{{1}}A_{{0}}\xi + ( ( \theta _{{2}}+q_{{0}} ) c_{{1}}m _{{N_{{q}}}}+qc_{{1}}t_{{3}} ) A_{{0}} ) t_{{4}}-\xi \,A_{{0 }}c_{{1}}h_{{3}}m_{{N_{{q}}}}-A_{{0}}c_{{1}}h_{{3}} ) q_{{2}}- ( h_{{2}}\xi \,B_{{1}}\\&+ \, t_{{4}}A_{{1}} ( \xi \,t_{{3}}\theta _{ {1}}+m_{{N_{{q}}}}q_{{0}} ) ) t_{{2}}c_{{0}}+B_{{1}} \theta _{{4}}-t_{{2}}t_{{4}}A_{{0}} ( \xi \,t_{{3}}\theta _{{1}}+m_{ {N_{{q}}}}q_{{0}} ) c_{{1}} ) q_{{5}}+ ( ( ( -\xi \,h_{{2}}+\theta _{{4}} ) B_{{1}}+t_{{4}}A_{{1}} ( \xi \,t_{{3}}\theta _{{1}}\\&+ \, m_{{N_{{q}}}}q_{{0}} ) )c_{{0}}+t_{{4}}A_{{0}} ( \xi \,t_{{3}}\theta _{{1}}+m_{{N_ {{q}}}}q_{{0}} ) c_{{1}} ) t_{{2}}\sigma _{{3}} ) q_{ {7}}-B_{{1}}t_{{2}}\sigma _{{3}}c_{{0}} ( \xi \,h_{{2}}-\theta _{{4} } ) ) q_{{4}}-q_{{2}}\sigma _{{2}}(( -B_{{1}} q_{{5}}-\theta _{{5}}B_{{1}} ) q_{{7}}-B_{{1}}\\&( q_{{5}} \epsilon _{{1}}+\theta _{{5}}\epsilon _{{2}}))) q_ {{3}}+q_{{2}}q_{{4}}q_{{5}}q_{{7}}\sigma _{{1}}B_{{1}} ) q_{{6}}+ q_{{2}}q_{{4}}q_{{5}}q_{{7}}\sigma _{{1}}\epsilon _{{3}}B_{{1}}+ \frac{-B_{2}q_{2}q_{5}q_{6}q_{7}}{1+\theta _{3}}\,({q _{3}}^2{q_{4}}^2R_{E_{m}}+q_{1}q_{3}q_{4}R_{I_{a}}+q_{1}q_{3}\\&q_{4}R_{I_{m}}),\\ \end{aligned}} \\ & {\begin{aligned} C_{2}=&-q_{{3}}q_{{6}}t_{{4}}\epsilon _{{2}}A_{{2}}q_{{4}} ( \xi \,t_{{3}} \theta _{{1}}+m_{{N_{{q}}}}q_{{0}})+((( q_{2}-t_{2}) q_{{5}}-t_{{2}}\sigma _{{3}})q_{{7}}-t_{{2}}\sigma _{{3}} ) q_{{3}}\xi \,h_{{1}}q_{{4}}( B_{{1}}c_{{1}}+B_{{2}}c_{{0}}) {q_{{6}}}^{2} \\ {}&+ \, (((((((( (qh_{{1}}+h_{{2}}( q+1))B_{{2}}+A_{{2}}t_{{3}}t_{{4}}\theta _{{1}} +\theta _{{3}} ( h_{{1}} +h_{{2}} ) B_{{1}}-h_{{3}}m_{{N_{q}}}A_{{2}} ) c_{{0}}+( A_{1}t_{{3}}t_{{4}}\theta _{1}+(qh_{1} \\&+ \, h_{2}( q+1))B_{1} -h_{{3}}m_{{N_{q}}}A_{1}) c_{{1}} ) \xi + ( h_{{1}} ( qq_{{0}}-\theta _{{1}}\theta _{{2}} ) B_{{2}}+ ( t_{{3}} \theta _{{3}}A_{{1}}+ ( ( \theta _{{2}}+q_{{0}} ) m_{{N_{{q}}}}+qt_{{3}} ) A_{{2}} ) t_{{4}} \\&+ \, \theta _{{3}}q_{{0}}h _{{1}}B_{{1}}-h_{{3}}A_{{2}} ) c_{{0}}+ ( ( ( ( \theta _{{2}}+q_{{0}} ) m_{{N_{{q}}}}+qt_{{3}} ) A_{{1}}+t_{{3}}\theta _{{3}}A_{{0}} ) t_{{4}}+h_{{1}} ( qq_{{0 }}-\theta _{{1}}\theta _{{2}} ) B_{{1}}-h_{{3}}A_{{1}} ) c_{ {1}}\\&+ \, B_{{2}} ) q_{{2}} -(( A_{{2}}t_{{3}}t_{{4}} \theta _{{1}}+B_{{2}}h_{{2}} ) c_{{0}}+c_{{1}} ( A_{{1}}t_{{ 3}}t_{{4}}\theta _{{1}}+B_{{1}}h_{{2}} ) ) t_{{2}}\xi -c_{{ 1}}t_{{2}}t_{{4}}m_{{N_{{q}}}}q_{{0}}A_{{1}}-t_{{2}}t_{{4}}m_{{N_{{q}} }}q_{{0}}A_{{2}}c_{{0}} \\& + \, B_{{2}}\theta _{{4}} ) q_{{5}}-\sigma _{{3 }}t_{{2}} ( ( ( -A_{{2}}t_{{3}}t_{{4}}\theta _{{1}}+B_ {{2}}h_{{2}} ) c_{{0}}+c_{{1}} ( -A_{{1}}t_{{3}}t_{{4}} \theta _{{1}}+B_{{1}}h_{{2}} ) ) \xi + ( -t_{{4}}m_{{N _{{q}}}}q_{{0}}A_{{2}}-B_{{2}}\theta _{{4}} ) c_{{0}}\\&- \, c_{{1}} ( t_{{4}}m_{{N_{{q}}}}q_{{0}}A_{{1}}+B_{{1}}\theta _{{4}} ) ) ) q_{{7}}-t_{{2}}\sigma _{{3}} ( \xi \,h_{ {2}}-\theta _{{4}} ) ( B_{{1}}c_{{1}}+B_{{2}}c_{{0}} ) ) q_{{4}}+B_{{2}} ( ( q_{{5}}+\theta _{{5}} ) q_{{7}}+q_{{5}}\epsilon _{{1}}+\theta _{{5}}\epsilon _{{2}} )\\&q_{{2}}\sigma _{{2}} ) q_{{3}} +q_{{2}}q_{{4}}q_{{5}}q_{{ 7}}\sigma _{{1}}B_{{2}} ) q_{{6}}+q_{{2}}q_{{4}}q_{{5}}q_{{7}} \sigma _{{1}}\epsilon _{{3}}B_{{2}},\\ \end{aligned}} \\ & {\begin{aligned} C_{3}=&((((((((q+q_{{6}} )h_{{1}}+h_{{2}}(q+1 )) B_{{2}} +(t_{{3}}\theta _{{1}}t_{{4}}-h_{{3}}m_{{N_{{q}}}} )A_{{2}}+\theta _{{3}} ( h_{{1}}+h_{{2}} ) B_{{1}} ) \xi +h_{{1}} ( qq_{{0}}-\theta _{{1}}\theta _{{2}} ) B_{{2}} \\&+ \, ( ( ( \theta _{{2}}+q_{{0}} ) m_{{N_{{q}}}}+qt_{{3}} ) t_{{4}}-h_{{3}} ) A_{{2}}+\theta _{{3}} ( A_{{1}}t_ {{3}}t_{{4}}+B_{{1}}h_{{1}}q_{{0}} ) ) q_{{2}}- ( ( ( h_{{1}}q_{{6}}+h_{{2}} ) B_{{2}}+A_{{2}}t_{{3}}t _{{4}}\theta _{{1}} ) \xi \\& + \, t_{{4}}m_{{N_{{q}}}}q_{{0}}A_{{2}} ) t_{{2}} ) q_{{5}}- ( ( ( h_{{1}}q_{{6} }+h_{{2}} ) B_{{2}}-A_{{2}}t_{{3}}t_{{4}}\theta _{{1}} ) \xi -t_{{4}}m_{{N_{{q}}}}q_{{0}}A_{{2}}-B_{{2}}\theta _{{4}} ) \sigma _{{3}}t_{{2}} ) c_{{1}}+\theta _{{3}}q_{{2}}q_{{5}} ( B_{{2}} ( h_{{1}}+h_{{2}} ) \xi \\&+ \, q_{{0}}h_{{1}}B_{{2}}+t_{{3 }}t_{{4}}A_{{2}} ) c_{{0}} ) q_{{7}}-B_{{2}} ( ( h_{{1}}q_{{6}}+h_{{2}} ) \xi -\theta _{{4}} ) \sigma _{{3}}c_{{1}}t_{{2}} ) q_{{6}}q_{{4}}q_{{3}},\\ \end{aligned}} \end{aligned}$$

and

$$\begin{aligned} \begin{aligned} C_{4}=q_{{2}} ( ( ( \xi +q_{{0}} ) h_{{1}}+\xi \,h_{{2} } ) B_{{2}}+t_{{3}}t_{{4}}A_{{2}} ) q_{{7}}q_{{5}}\theta _{ {3}}q_{{3}}q_{{6}}c_{{1}}q_{{4}}.\\ \end{aligned} \end{aligned}$$

To be more specific and discuss the possible of roots of Eq. (21), we used Descartes’ rule of sign changes^46^ and showed 32 possible results in Table 4.

**Table 4:** Number of possible positive real roots of Eq. (21).

Case	$C_{4}$	$C_{3}$	$C_{2}$	$C_{1}$	$C_{0}$	${\mathscr {R}}_{0}$	No. of possible changes	Positive Real Roots
1	–	–	–	–	–	${\mathscr {R}}_{0}>1$	0	0
2	–	$+$	$+$	$+$	$+$	${\mathscr {R}}_{0}<1$	1	1
3	–	–	–	–	$+$	${\mathscr {R}}_{0}<1$	1	1
4	–	–	–	$+$	$+$	${\mathscr {R}}_{0}<1$	1	1
5	–	–	–	$+$	$+$	${\mathscr {R}}_{0}<1$	1	1
6	–	–	$+$	$+$	$+$	${\mathscr {R}}_{0}<1$	1	1
7	–	$+$	$+$	$+$	–	${\mathscr {R}}_{0}>1$	2	0,2
8	–	$+$	–	–	–	${\mathscr {R}}_{0}>1$	2	0,2
9	–	–	$+$	–	–	${\mathscr {R}}_{0}>1$	2	0,2
10	–	–	–	$+$	–	${\mathscr {R}}_{0}>1$	2	0,2
11	–	$+$	$+$	–	–	${\mathscr {R}}_{0}>1$	2	0,2
12	–	–	$+$	$+$	–	${\mathscr {R}}_{0}>1$	2	0,2
13	–	–	$+$	–	$+$	${\mathscr {R}}_{0}<1$	3	1,3
14	–	$+$	$+$	–	$+$	${\mathscr {R}}_{0}<1$	3	1,3
15	–	$+$	–	$+$	$+$	${\mathscr {R}}_{0}<1$	3	1,3
16	–	$+$	–	$+$	−	${\mathscr {R}}_{0}>1$	4	0,2,4
17	$+$	$+$	$+$	$+$	$+$	${\mathscr {R}}_{0}<1$	0	0
18	$+$	$+$	$+$	$+$	–	${\mathscr {R}}_{0}>1$	1	1
19	$+$	–	–	–	–	${\mathscr {R}}_{0}>1$	1	1
20	$+$	$+$	−	−	−	${\mathscr {R}}_{0}>1$	1	1
21	$+$	$+$	$+$	–	–	${\mathscr {R}}_{0}>1$	1	1
22	$+$	–	–	–	$+$	${\mathscr {R}}_{0}<1$	2	0,2
23	$+$	–	–	$+$	$+$	${\mathscr {R}}_{0}<1$	2	0,2
24	$+$	–	–	$+$	$+$	${\mathscr {R}}_{0}<1$	2	0,2
25	$+$	$+$	$+$	–	$+$	${\mathscr {R}}_{0}<1$	2	0,2
26	$+$	$+$	–	$+$	$+$	${\mathscr {R}}_{0}<1$	2	0,2
27	$+$	–	$+$	$+$	$+$	${\mathscr {R}}_{0}<1$	2	0,2
28	$+$	–	$+$	–	–	${\mathscr {R}}_{0}>1$	3	1,3
29	$+$	–	–	$+$	–	${\mathscr {R}}_{0}>1$	3	1,3
30	$+$	$+$	–	$+$	–	${\mathscr {R}}_{0}>1$	3	1,3
31	$+$	–	$+$	$+$	–	${\mathscr {R}}_{0}>1$	3	1,3
32	$+$	–	$+$	–	$+$	${\mathscr {R}}_{0}<1$	4	0,2,4

### Fitting analysis

We inputted into the model the data from 24 January to 31 October 2020 in Hong Kong by employing the Pearson’s Chi-squared test and the least square method via **R** statistical software version 3.4.1^47^. The demographic related data includes $\pi$ as natural birth 225 and $\mu _{H}$ as the crude death rate 0.00003^29^. The initial $S_{0}$ is set as 7,181,657 on 24th January 2020, which equals the summation of the net growth of inbound travellers^28^, initial local population 7,520,800^42^ and released quarantined inbound travellers. With reference to the epidemic model in Eq. (1) and local data, we assume $m_{N}$ as zero since the number of inbound travellers without quarantine is unknown. All inbound travellers are assumed to follow quarantine rules since 24 January 2020. During the first 13 days, no quarantined visitor is released: $m_{N_{q}}(1)=...=m_{N_{q}}(13)=0$. All initial values of parameters are shown in Table 2.

The Hong Kong government announced a quarantine policy on 25 March 2020. All inbound travellers are required to quarantine for 14 days after arriving in Hong Kong. During quarantine, people are not allowed to have any close contact with others. If an inbound traveller becomes symptomatic, the traveller will be hospitalized by the government. If the latency period exceeds 14 days, the released quarantine people can still transmit the virus to others as asymptomatic infectious individuals; owing to the absence of symptoms, it is difficult to screen them from the public. As a result, the Hong Kong government has imposed various restrictions designed to reduce the risk of transmission among the whole population, such as one-meter social distancing^48^, wearing masks, a ban on restaurant dining, and restrictions on the maximum number of people gathering. The proposed ‘effective contact ratio’ helps describe the degree of adherence to these restrictions among the public. Based on six types used by the Hong Kong government^17^, we subdivide confirmed cases based on the infection sources and symptoms. Imported cases and cases epidemiologically linked with imported cases are from or caused by quarantined individuals. The other four types related to local cases are divided into asymptomatic or symptomatic cases. Infection control policies enacted by the Hong Kong government are indicated in Fig. 4.

## Supplementary Information

Supplementary Information 1.

## Data Availability

Data on COVID-19 was acquired from the Centre for Health Protection (CHP) of the Department of Health, the Government of the Hong Kong Special Administrative Region. These data sources are freely accessible through web-based archives. All data has been collected via GitHub (https://github.com/YUANZIYUE1997/covid19).

## References

[1] WHO. World Health Organization (WHO) Coronavirus Disease (COVID-19) Dashboard. https://covid19.who.int/ (2021). Accessed 15 Mar 2021.

[2] ECDC, European Centre for Disease Prevention and Control. COVID-19 situation update for the EU and the UK (as of 20 November 2020). https://www.ecdc.europa.eu/en/cases-2019-ncov-eueea (2020). Accessed 21 Nov 2020.

[3] Bridle, B. W. 5 factors that could dictate the success or failure of the covid-19 vaccine rollout. https://theconversation.com/5-factors-that-could-dictate-the-success-or-failure-of-the-covid-19-vaccine-rollout-152856 (2021). Accessed 3 Apr 2021.

[4] World Health Organization. Pandemic fatigue: Reinvigorating the public to prevent covid-19: Policy framework for supporting pandemic prevention and management: Revised version November 2020 (no. who/euro: 2020-1573-41324-56242). World Health Organization. Regional office for Europe. https://www.euro.who.int/en/health-topics/health-determinants/behavioural-and-cultural-insights-for-health/publications/2020/pandemic-fatigue-reinvigorating-the-public-to-prevent-covid-19,-september-2020-produced-by-whoeurope (2020). Accessed 2 Dec 2020.

[5] Kermack WO, McKendrick AG (1927). A contribution to the mathematical theory of epidemics. Proc. R. Soc. Lond. Ser. A Contain. Pap. Math. Phys. Char..

[6] Wu JT, Leung K, Leung GM (2020). Nowcasting and forecasting the potential domestic and international spread of the 2019-nCoV outbreak originating in Wuhan, China: A modelling study. Lancet.

[7] Tang B (2020). Estimation of the transmission risk of the 2019-nCoV and its implication for public health interventions. J. Clin. Med..

[8] Lin Q (2020). A conceptual model for the coronavirus disease 2019 (COVID-19) outbreak in Wuhan, China with individual reaction and governmental action. Int. J. Infect. Dis..

[9] Lau H (2020). The association between international and domestic air traffic and the coronavirus (COVID-19) outbreak. J. Microbiol. Immunol. Infect..

[10] Hu M (2021). Risk of coronavirus disease 2019 transmission in train passengers: An epidemiological and modeling study. Clin. Infect. Dis..

[11] Mo B (2021). Modeling epidemic spreading through public transit using time-varying encounter network. Transport. Res. Part C Emerg. Technol..

[12] Safi MA, Gumel AB (2013). Dynamics of a model with quarantine-adjusted incidence and quarantine of susceptible individuals. J. Math. Anal. Appl..

[13] Ngonghala CN (2020). Mathematical assessment of the impact of non-pharmaceutical interventions on curtailing the 2019 novel coronavirus. Math. Biosci..

[14] Musa SS (2020). Mechanistic modelling of the large-scale lassa fever epidemics in Nigeria from 2016 to 2019. J. Theor. Biol..

[15] Wong DW, Li Y (2020). Spreading of COVID-19: Density matters. PLoS One.

[16] Hale T (2021). A global panel database of pandemic policies (Oxford covid-19 government response tracker). Nat. Hum. Behav..

[17] Centre for Health Protection. Latest situation of coronavirus disease (COVID-19) in Hong Kong. https://chp-dashboard.geodata.gov.hk/covid-19/en.html. Accessed 4 Apr 2021.

[18] He X (2020). Temporal dynamics in viral shedding and transmissibility of COVID-19. Nat. Med..

[19] Hens N (2010). Seventy-five years of estimating the force of infection from current status data. Epidemiol. Infect..

[20] Tang X, Musa SS, Zhao S, He D (2021). Reinfection or reactivation of severe acute respiratory syndrome coronavirus 2: A systematic review. Front. Public Health.

[21] Callaway, E. Heavily mutated coronavirus variant puts scientists on alert. *Nature* (2021). Access 27 Nov 2021. 10.1038/d41586-021-03552-w 34824381

[22] Murray CJ, Piot P (2021). The potential future of the COVID-19 pandemic: Will SARS-CoV-2 become a recurrent seasonal infection?. JAMA.

[23] Ma X, Zhou Y, Cao H (2013). Global stability of the endemic equilibrium of a discrete SIR epidemic model. Adv. Differ. Equ..

[24] Van den Driessche P, Watmough J (2002). Reproduction numbers and sub-threshold endemic equilibria for compartmental models of disease transmission. Math. Biosci..

[25] Wu JT (2010). The infection attack rate and severity of 2009 pandemic H1N1 influenza in Hong Kong. Clin. Infect. Dis..

[26] Gao D (2016). Prevention and Control of Zika as a Mosquito-Borne and Sexually Transmitted Disease: A Mathematical Modeling Analysis. Sci. Rep..

[27] The MathWorks, Inc. SimBiology: SimBiology Model Builder and SimBiology Model Analyzer. https://www.mathworks.com/products/simbiology.html (2021).

[28] Immigration Department. Statistics on Passenger Traffic (January 2020). https://www.immd.gov.hk/eng/message_from_us/stat2.html. Accessed 15 Nov 2020.

[29] Hong kong life expectancy 1950-20200. https://www.macrotrends.net/countries/HKG/hong-kong/life-expectancy. Retrieved 2020-12-16.

[30] Britton T, Ball F, Trapman P (2020). A mathematical model reveals the influence of population heterogeneity on herd immunity to SARS-CoV-2. Science.

[31] Wallinga J, Teunis P, Kretzschmar M (2006). Using data on social contacts to estimate age-specific transmission parameters for respiratory-spread infectious agents. Am. J. Epidemiol..

[32] YUAN, Z. Local data related COVID-19 in Hong Kong (updated till 30th November 2020). https://github.com/YUANZIYUE1997/covid19.

[33] Mu X, Yeh AG-O, Zhang X (2020). The interplay of spatial spread of COVID-19 and human mobility in the urban system of china during the Chinese new year. Environ. Plan. B Urban Anal. City Sci..

[34] Mass Transit Railway (MTR). Historic patronage figues from Jan. 2020 to Dec. 2020. https://www.mtr.com.hk/en/corporate/investor/patronage.php#search. Accessed 18 Jan 2022.

[35] Gumel AB (2012). Causes of backward bifurcations in some epidemiological models. J. Math. Anal. Appl..

[36] Phil Mercer. Hotel quarantine under scrutiny as australian state races to contain COVID-19 outbreak. https://www.voanews.com/covid-19-pandemic/hotel-quarantine-under-scrutiny-australian-state-races-contain-covid-19-outbreak. Accessed 5 Mar 2021.

[37] Government of Hong Kong. Government further strengthens compulsory testing. https://www.info.gov.hk/gia/general/202102/03/P2021020300018.htm?fontSize=1. Accessed 1 Mar 2021.

[38] Victor Ting. Hong kong fourth wave: Sewage tests for coronavirus to be expanded, aim for ‘gold standard’. https://www.scmp.com/news/hong-kong/health-environment/article/3117041/hong-kong-fourth-wave-sewage-tests-coronavirus-be?utm_source=copy_link&utm_medium=share_widget&utm_campaign=3117041. Accessed 1 Mar 2021.

[39] K11 diner cluster grows to 34 as HK logs 33 new cases. https://www.thestandard.com.hk/breaking-news/section/4/166385/K11-diner-cluster-grows-to-34-as-HK-logs-33-new-cases. Accessed 1 Mar 2021.

[40] Guan W-J (2020). Clinical characteristics of coronavirus disease 2019 in China. N. Engl. J. Med..

[41] Iwasaki A (2021). What reinfections mean for COVID-19. Lancet. Infect. Dis.

[42] Census and Statistic Department. Population estimates. https://www.censtatd.gov.hk/hkstat/sub/sp150.jsp?tableID=001&ID=0&productType=8. Accessed 10 Nov 2020.

[43] Anguelov R, Garba SM, Usaini S (2014). Backward bifurcation analysis of epidemiological model with partial immunity. Comput. Math. Appl..

[44] Musa SS (2019). A mathematical model to study the 2014–2015 large-scale dengue epidemics in Kaohsiung and Tainan cities in Taiwan, China. Math. Biosci. Eng..

[45] Hussaini N, Okuneye K, Gumel AB (2017). Mathematical analysis of a model for zoonotic visceral leishmaniasis. Infect. Dis. Modell..

[46] Ghosh I, Tiwari PK, Chattopadhyay J (2019). Effect of active case finding on dengue control: Implications from a mathematical model. J. Theor. Biol..

[47] RStudio Team (2015). RStudio: Integrated Development Environment for R.

[48] 7 ways to fight the virus under the new normal: 3. maintain social distancing. https://www.coronavirus.gov.hk/eng/7-ways-fight.html (2020). Accessed 16 Dec 2020.

