# Supplementary material for: Post pandemic fatigue: what are effective strategies?
Source: Sci Rep. 2022 Jun 11;12:9706. doi: 10.1038/s41598-022-13597-0 (PMC9188281; doi:10.1038/s41598-022-13597-0)
Supplement: Supplementary file 1 — Supplementary Information 1. [file 41598_2022_13597_MOESM1_ESM.pdf]

Supplementary Information
Post Pandemic Fatigue: What are Effective Strategies?

Ziyue YUAN[1], Salihu Sabiu Musa[2,3], Shu-Chien Hsu[1,*], Clara Man Cheung[4], and Daihai He[2]

[1]Department of Civil and Environmental Engineering, Hong Kong Polytechnic University, Hong Kong
[2]Department of Applied Mathematics, Hong Kong Polytechnic University, Hong Kong
[3]Department of Mathematics, Kano University of Science and Technology, Wudil, Nigeria
[4]Department of Mechanical, Aerospace and Civil Engineering, School of Engineering, University of Manchester, United Kingdom
[*]mark.hsu@polyu.edu.hk

Supplementary Information

SI.1 Data description

The time-series data of COVID-19 confirmed cases were obtained from Hong Kong Centre for the Health Protection (CHP) in the period between 24th January 2020 and 13th April 2021[1], i.e., the period during which Immigration Department recorded the Statistics on Passenger Traffic[2]. From the demography related data in Table SI.1, the transmission dynamics of SARS-CoV-2 is not gender or age dependent[3]. We assume that every individual should have an identical possibility of getting infected with the same risk of death. The median of the generation interval (GI) for COVID-19 was estimated as five days[4], but it can be longer than seven days[5]. We notice about three percent of confirmed cases in Hong Kong have an exceeded-14-day time-delay between symptom onset and reporting, reflecting the possibility of indirectly showing the symptom after quarantine. Additionally, more than 30 percent of infectious individuals are asymptomatic. This study considers both asymptomatic cases and people who onset after quarantine.

SI.2 The basic reproduction number

Applying the next generation method[6] to the equations in model (1), the new infections F and transition terms V are as follow: To analyze the interplay between different groups, we defined a_1, a_2, a_3, a_4, a_5 and a_6 as the effective contact ratio between E_m and S, I_a and S, I_m and S,E_m and N_q, I_a and N_q and I_m and N_q. The transmission rates for S and N_q are assumed as β_1 and β_2 respectively. Thus, F can be rewritten as:

$$F = \begin{bmatrix} \beta_1 a_1 + \theta_3 \beta_2 a_4 & 0 & \beta_1 a_2 + \theta_3 \beta_2 a_5 & \beta_1 a_3 + \theta_3 \beta_2 a_6 & 0 & 0 & 0 \\ 0 & 0 & 0 & 0 & 0 & 0 & 0 \\ 0 & 0 & 0 & 0 & 0 & 0 & 0 \\ 0 & 0 & 0 & 0 & 0 & 0 & 0 \\ 0 & 0 & 0 & 0 & 0 & 0 & 0 \\ 0 & 0 & 0 & 0 & 0 & 0 & 0 \\ 0 & 0 & 0 & 0 & 0 & 0 & 0 \end{bmatrix}.$$

Table SI.1. **Local Situation of COVID-19 in Hong Kong(as of April 13, 2021)**

**Symptom Type**	**Subtotal**	**Percentage**
Symptomatic	8087	69.75
Asymptomatic	3508	30.25
**Gender Distribution**	**Subtotal**	**Percentage**
Female	6008	50.88
Male	5587	49.12
**Age Distribution**	**Subtotal**	**Percentage**
$(0,10]$	548	4.96
$(10,20]$	762	6.90
$(20,30]$	1752	15.86
$(30,40]$	2073	18.77
$(40,50]$	1891	17.12
$(50,60]$	1852	16.76
$(60,70]$	1649	14.93
$(70,80]$	683	6.18
$(80,90]$	316	2.86
$(90,120]$	69	0.62
**Distribution of Time-delay between Symptom Onset and Reporting**	**Subtotal**	**Percentage**
$(-3,0]$	21	0.26
$(0,5]$	5628	69.77
$(5,10]$	1889	23.42
$(10,15]$	404	5.01
$(15,20]$	100	1.24
$(20,30]$	33	0.41
$(30,40]$	9	0.11
$(40,100]$	3	0.04
**Fatality Rate distributed based on Age**	**Subtotal**	**Percentage**
$(0,35]$	0	0.00
$(35,60]$	17	0.35
$(60,70]$	30	1.82
$(70,80]$	56	8.20
$(80,85]$	42	22.34
$(85,90]$	40	31.25
$(90,120]$	22	31.88

The transition terms V is as follows:

$$V = \begin{bmatrix} q_1 & 0 & 0 & 0 & 0 & 0 & 0 \\ -\theta_4 & q_2 & 0 & 0 & 0 & 0 & 0 \\ -\sigma_1 & 0 & q_3 & 0 & 0 & 0 & 0 \\ -\sigma_2 & 0 & 0 & q_4 & 0 & 0 & 0 \\ 0 & -\sigma_3 & 0 & -\theta_5 & q_5 & 0 & 0 \\ 0 & 0 & -\varepsilon_3 & 0 & 0 & q_6 & 0 \\ 0 & 0 & 0 & -\varepsilon_1 & -\varepsilon_2 & 0 & q_7 \end{bmatrix}.$$

SI.3 The Proof of the Existence of EE

Given the model (1), we obtain

$$N_q^* = \frac{m_{N_q} + \theta_1 S^*}{q_0 + \theta_3 \lambda^*} \tag{SI.1}$$

$$E_m^* = \frac{(\theta_3 {\lambda^*}^2 + (q_0 + \theta_1 \theta_3)\lambda^*) S^* + \theta_3 m_{N_q} \lambda^*}{q_1 (q_0 + \theta_3 \lambda^*)} \tag{SI.2}$$

$$I_a^* = \frac{\sigma_1(\theta_3 {\lambda^*}^2 + (q_0 + \theta_1 \theta_3)\lambda^*)}{q_3 q_1 (q_0 + \theta_3 \lambda^*)} S^* + \frac{\sigma_1(\theta_3 {\lambda^*}^2 + (q_0 + \theta_1 \theta_3)\lambda^*)}{q_3 q_1 (q_0 + \theta_3 \lambda^*)} \tag{SI.3}$$

$$I_m^* = \frac{\sigma_2(\theta_3 {\lambda^*}^2 + (q_0 + \theta_1 \theta_3)\lambda^*)}{q_4 q_1 (q_0 + \theta_3 \lambda^*)} S^* + \frac{\sigma_2 \theta_3 m_{N_q} \lambda^*}{q_4 q_1 (q_0 + \theta_3 \lambda^*)} \tag{SI.4}$$

$$H_a^* = \frac{\varepsilon_3 \sigma_1(\theta_3 {\lambda^*}^2 + (q_0 + \theta_1 \theta_3)\lambda^*)}{q_3 q_6 q_1 (q_0 + \theta_3 \lambda^*)} S^* + \frac{\varepsilon_3 \sigma_1 \theta_3 m_{N_q} \lambda^*}{q_3 q_6 q_1 (q_0 + \theta_3 \lambda^*)} \tag{SI.5}$$

$$E_q^* = \frac{\theta_1 q_1 (1 - \theta_2 - \theta_3) + \theta_4(\theta_3 {\lambda^*}^2 + (q_0 + \theta_1 \theta_3)\lambda^*)}{q_2 q_1 (q_0 + \theta_3 \lambda^*)} S^* + \frac{m_{N_q} q_1 (1 - \theta_2 - \theta_3) + \theta_4 \theta_3 m_{N_q} \lambda^*}{q_2 q_1 (q_0 + \theta_3 \lambda^*)} \tag{SI.6}$$

$$\begin{aligned} H_s^* =& [\frac{(\varepsilon_1 q_5 + \varepsilon_2 \theta_5)\, \sigma_2(\theta_3 {\lambda^*}^2 + (q_0 + \theta_1 \theta_3)\lambda^*)}{q_4 q_5 q_7 q_1 (q_0 + \theta_3 \lambda^*)} + \frac{\theta_1 q_1 \varepsilon_2 \sigma_3 (1 - \theta_2 - \theta_3) + \varepsilon_2 \sigma_3 \theta_4 (\theta_3 {\lambda^*}^2 + (q_0 + \theta_1 \theta_3)\lambda^*)}{q_2 q_7^2 q_1 (q_0 + \theta_3 \lambda^*)}] S^* + \\ & [\frac{(\varepsilon_1 q_5 + \varepsilon_2 \theta_5)\, \sigma_2 \theta_3 m_{N_q} \lambda^*}{q_4 q_5 q_7 q_1 (q_0 + \theta_3 \lambda^*)} + \frac{\varepsilon_2 \sigma_3 [q_1 (1 - \theta_2 - \theta_3) m_{N_q} + \theta_4 \theta_3 m_{N_q} \lambda^*}{q_2 q_7^2 q_1 (q_0 + \theta_3 \lambda^*)}] \end{aligned} \tag{SI.7}$$

$$\begin{aligned} I_q^* =& \frac{\theta_1 \sigma_3 q_1 q_4 (1 - \theta_2 - \theta_3) + \sigma_3 \theta_4 q_4 (\theta_3 {\lambda^*}^2 + (q_0 + \theta_1 \theta_3)\lambda^*) + \theta_2 \sigma_2 q_2 (\theta_3 {\lambda^*}^2 + (q_0 + \theta_1 \theta_3)\lambda^*)}{q_2 q_4 q_5 q_1 (q_0 + \theta_3 \lambda^*)} S^* \\ & + \frac{\sigma_3 q_1 q_4 m_{N_q} (1 - \theta_2 - \theta_3) + \sigma_2 \theta_5 q_2 \theta_3 m_{N_q} \lambda^*}{q_2 q_4 q_5 q_1 (q_0 + \theta_3 \lambda^*)} \end{aligned} \tag{SI.8}$$

From above, we can easily get $N_q^* \ldots ,I_q^*$ are positive with a non-negative S^*.
Substitute Eq. (SI.1), (SI.3), (SI.4), (SI.8), (SI.7) and (SI.5) into (1), $\frac{dR}{dt}$ can be rewritten as below:

$$R^* = \frac{\gamma_1 I_a^* + \gamma_2 I_m^* + \gamma_3 I_q^* + \gamma_4 H_s^* + \gamma_5 H_a^*}{q_8} \tag{SI.9}$$

Obviously, R^* is larger than zero when $I_a^* \ldots ,H_a^*$ are positive. Hence, the EE exits in terms of a positive solution of model (1).

SI.4 The initial variables and estimated parameters for four phases

All parameter descriptions and ranges are described in Table 2. Based on the force of infections in Eqns. (2), Table SI.2 describe the initial values of variables and estimated values of some parameters (i.e., β_1, β_2, θ_3, a_1, a_2, a_3, a_4, and a_5) for each phase.

	Phase 1	Phase 2	Phase 3	Phase 4
**Initial values of variables**				
S	7181657	7181657	8019956	8014725
N_q	162336	162336	34584	18185
E_m	300	1000	71	2527
E_q	15	20	8313	8779
I_a	16	270	557	748
I_m	5	170	108	2096
I_q	2	100	575	588
H_a	1	100	517	695
H_s	1	50	1419	2384
R	5	15	1313	1685
**Estimated values of parameters**				
β_1	0.6779	0.3856	1.0058	0.1518
β_2	0.1472	1.5234	0.9399	0.4864
θ_3	0.1596	0.4114	0.3134	0.3274
a_1	0.0298	0.0735	0.1981	0.1800
a_2	0.1076	0.1280	0.1912	0.1738
a_3	0.0575	0.1329	0.1968	0.1898
a_4	0.0445	0.1752	0.1049	0.1423
a_5	0.1072	0.1971	0.1208	0.1615

Table SI.2. The initial variables and estimated parameters for four phases (Phase 1: 24th Jan.-24th Mar., Phase 2: 25th May.-19th Jul., Phase 3: 20th Jul.-29th Jul. and Phase 4: 30th Jul.-31st Oct.).

References

1. Centre for Health Protection. Latest situation of coronavirus disease (COVID-19) in hong kong. https://chp-dashboard.geodata.gov.hk/covid-19/en.html. Accessed April 14, 2021.
2. Immigration Department. Statistics on Passenger Traffic (January 2020). https://www.immd.gov.hk/eng/message_from_us/stat2.html. Accessed November 15, 2020.
3. Jones, T. C. *et al.* An analysis of sars-cov-2 viral load by patient age. *MedRxiv* DOI: https://doi.org/10.1101/2020.06.08.20125484 (2020).
4. Griffin, J. *et al.* Rapid review of available evidence on the serial interval and generation time of covid-19. *BMJ open* **10**, e040263, DOI: http://dx.doi.org/10.1136/bmjopen-2020-040263 (2020).
5. Tang, X., Musa, S. S., Zhao, S., Mei, S. & He, D. Using proper mean generation intervals in modeling of covid-19. *Front. public health* **9** (2021).
6. Van den Driessche, P. & Watmough, J. Reproduction numbers and sub-threshold endemic equilibria for compartmental models of disease transmission. *Math. biosciences* **180**, 29–48, DOI: https://doi.org/10.1016/S0025-5564(02)00108-6 (2002).
